# Inferring ‘weak spots’ in phylogenetic trees: application to mosasauroid nomenclature

**DOI:** 10.7717/peerj.3782

**Published:** 2017-09-15

**Authors:** Daniel Madzia, Andrea Cau

**Affiliations:** 1 Institute of Paleobiology, Polish Academy of Sciences, Warsaw, Poland; 2 Department of Earth, Life and Environmental Sciences, Alma Mater Studiorum, University of Bologna, Bologna, Italy; 3 Geological and Paleontological Museum “G. Capellini”, Bologna, Italy

**Keywords:** Mosasauroidea, Phylogeny, Parsimony analysis, Bayesian inference, Phylogenetic nomenclature, Fossilized birth-death model, Late Cretaceous

## Abstract

Mosasauroid squamates represented the apex predators within the Late Cretaceous marine and occasionally also freshwater ecosystems. Proper understanding of the origin of their ecological adaptations or paleobiogeographic dispersals requires adequate knowledge of their phylogeny. The studies assessing the position of mosasauroids on the squamate evolutionary tree and their origins have long given conflicting results. The phylogenetic relationships within Mosasauroidea, however, have experienced only little changes throughout the last decades. Considering the substantial improvements in the development of phylogenetic methodology that have undergone in recent years, resulting, among others, in numerous alterations in the phylogenetic hypotheses of other fossil amniotes, we test the robustness in our understanding of mosasauroid beginnings and their evolutionary history. We re-examined a data set that results from modifications assembled in the course of the last 20 years and performed multiple parsimony analyses and Bayesian tip-dating analysis. Following the inferred topologies and the ‘weak spots’ in the phylogeny of mosasauroids, we revise the nomenclature of the ‘traditionally’ recognized mosasauroid clades, to acknowledge the overall weakness among branches and the alternative topologies suggested previously, and discuss several factors that might have an impact on the differing phylogenetic hypotheses and their statistical support.

## Introduction

Mosasauroidea was a species-rich clade of squamates adapted to an aquatic lifestyle, with evolutionary history being recorded exclusively in the Upper Cretaceous strata (e.g., Russell, 1967; Bell, 1997; Polcyn et al., 2014). During their history, mosasauroids distributed globally and evolved different ecological strategies (Polcyn et al., 2014; Bardet et al., 2015). Although the most distinguishable mosasauroid lineages, such as tylosaurines, plioplatecarpines, and derived mosasaurines, have been adequately recognized decades ago (Russell, 1967), the knowledge of mosasauroid origins and interrelationships is far from exhaustive. The studies of mosasauroid beginnings have long suffered from conflicting results of large-scale phylogenetic analyses (e.g., Lee, 1998; Conrad, 2008; Conrad et al., 2011; Gauthier et al., 2012), leading to the rise of considerable uncertainties surrounding the phylogenetic placement of Mosasauria within Squamata. However, recent analyses integrating morphological and molecular data show a good support for close relationships of Mosasauria and Serpentes within Toxicofera (Reeder et al., 2015).

The hypotheses of mosasauroid interrelationships appear to be less problematic. Phylogenetic studies frequently reconstruct the clades Halisaurinae, Mosasaurinae, Tylosaurinae, and Plioplatecarpinae (e.g., Bardet et al., 2005; Bell & Polcyn, 2005; Cuthbertson et al., 2007; Bullard & Caldwell, 2010; Fanti, Cau & Negri, 2014; Leblanc, Caldwell & Bardet, 2012; Jiménez-Huidobro & Caldwell, 2016), and a lineage of early-branching mosasaurids consisting of two branches that have recently been named Tethysaurinae and Yaguarasaurinae (Makádi, Caldwell & Ösi, 2012; Palci, Caldwell & Papazzoni, 2013; respectively).

Throughout the last two decades, the phylogenetic relationships within Mosasauroidea have been inferred using modified versions of a single data set. The data set was first introduced in Bell’s (1993) PhD thesis and formally published four years later (Bell, 1997). It was subsequently modified by inclusion of additional taxa and revision of characters and their states (see, e. g., Christiansen & Bonde, 2002; Dortangs et al., 2002; Bell & Polcyn, 2005; Polcyn & Bell, 2005; Bullard, 2006; Dutchak & Caldwell, 2006; Schulp, 2006; Schulp et al., 2006; Caldwell & Palci, 2007; Cuthbertson et al., 2007; Polcyn & Everhart, 2008; Dutchak & Caldwell, 2009; Fernandez & Martin, 2009; Leblanc, Caldwell & Bardet, 2012; Makádi, Caldwell & Ösi, 2012; Grigoriev, 2013; Palci, Caldwell & Papazzoni, 2013; Fanti, Cau & Negri, 2014; Jiménez-Huidobro & Caldwell, 2016; Otero et al., 2017; Simões et al., 2017).

The aim of this study is to estimate the robustness in our understanding of mosasauroid phylogenetic relationships by reevaluation of a recent version of that data set, published by Simões et al. (2017), that represents an effect of 20 years of detailed modifications regarding both taxon and character sampling. In particular, in this study we (1) focus on the implications of selection (or omission) among the tree-search strategies available for inferring phylogenetic relationships, a methodological bias that is often overlooked in phylogenetic systematics of fossil taxa, (2) revise the nomenclature of mosasauroid clades to assure that the applied clade names reflect differing tree topologies inferred by this and other studies, and to maintain the use of the names for the ‘traditional’ content, (3) discuss the factors that might have an impact on the differing phylogenetic hypotheses and their statistical support, and (4) suggest further modifications that may improve the resolution of the mosasauroid phylogenetic tree.

## Methods

Considering that all recent assessments of mosasauroid interrelationships are based on slightly modified versions of the same data set, we decided to refrain from incorporating substantial changes to the data of Simões et al. (2017) without extensive personal observations. Instead, we provide recommendations regarding further modifications and applied methodology (see ‘Discussion’).

However, slight modifications were provided regarding the binomial nomenclature. *Aigialosaurus bucchichi* was placed back within *Opetiosaurus* (following the potential non-monophyletic nature of the *dalmaticus*-*bucchichi* grouping, as inferred by some studies; e.g., Simões et al., 2017; our results), *Pannoniasaurus* ‘*osii*’ is ‘renamed’ *P. inexpectatus* (the original name established by Makádi, Caldwell & Ösi, 2012), *Halisaurus* ‘*sternbergi*’ is placed within *Eonatator*, as *E. sternbergii* (Bardet et al., 2005; Konishi et al., 2016), and *Platecarpus planifrons* is included within *Plesioplatecarpus* (Konishi & Caldwell, 2011).

We analyzed the data set under both, parsimony and Bayesian inference, the latter integrating morphological and stratigraphic data (using the method of Lee et al., 2014a, implemented by Lee et al., 2014b; Gavryushkina et al., 2017), to simultaneously infer topology and timing of evolutionary events (splitting of branches or placement of ancestors along lineages) of particular mosasauroid subclades. Note that Simões et al. (2017) performed both parsimony and Bayesian analyses, but did not integrate stratigraphic information in the Bayesian inference of their morphological data set.

### Parsimony analyses

Parsimony analyses were performed using TNT 1.5 (Goloboff, Farris & Nixon, 2008). In all analyses, we run 100 ‘New Technology’ search replicates, using default settings, saving all shortest trees inferred. Subsequently, for each analysis, we performed ‘Traditional Search’ heuristic search analyses exploring the tree islands inferred by the first round of analyses.

We performed three types of analyses: (1) with all characters having equal weight; first, keeping all multistate characters as unordered and, second, setting a subset of the multistate characters as ordered (listed below), (2) using the Implied Weighting option of TNT 1.5 (Goloboff, 1993; Goloboff, 1995; Goloboff et al., 2008) with three runs performed for both, ‘unordered’ and ‘ordered’ settings (*K* = 3, 6, and 9), and (3) with the same setting as in Simões et al. (2017) but using different ‘dolichosaur-grade’ taxa as sole outgroups.

**Figure 1:**
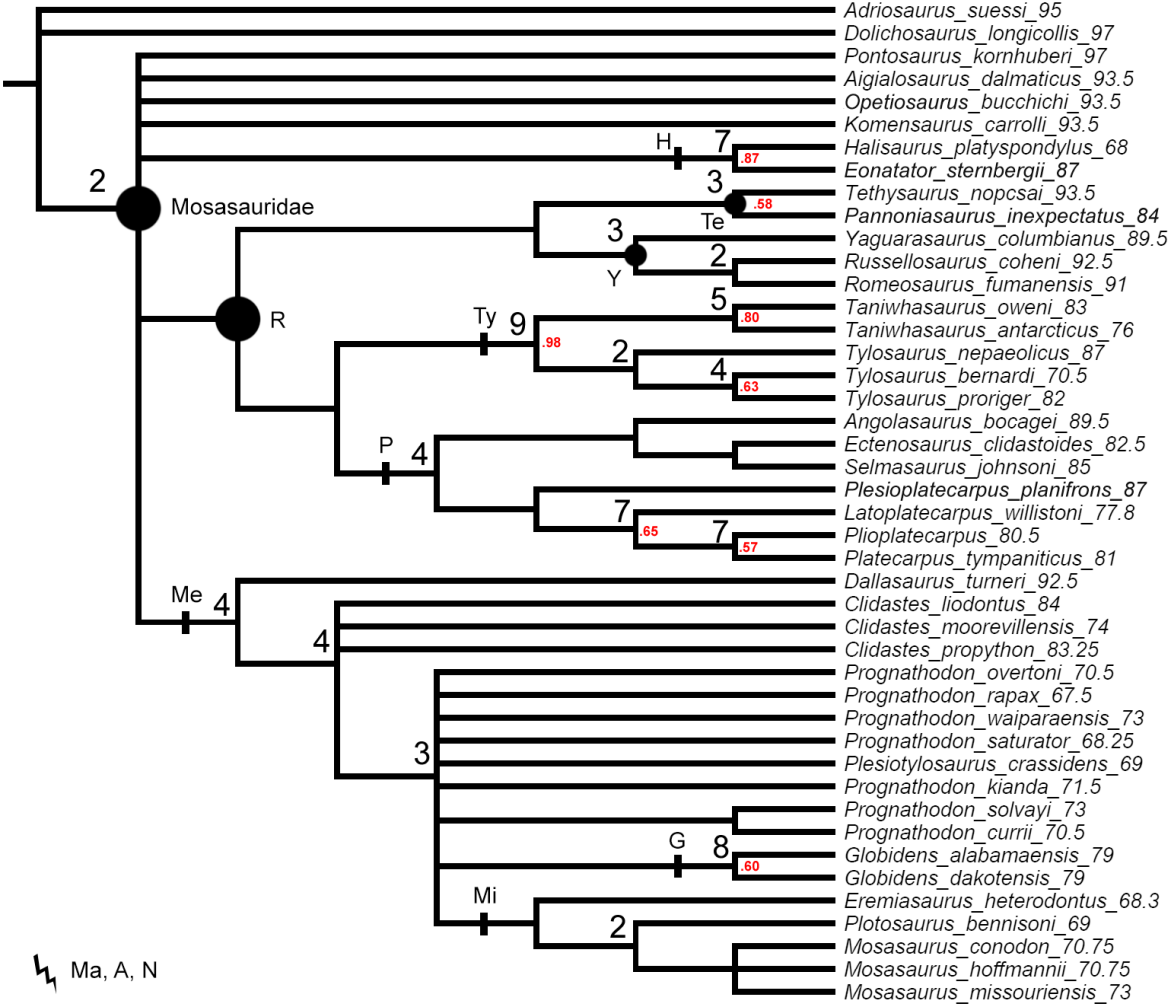
The strict consensus tree of 84 MPTs of length 445 inferred from unweighted parsimony analysis with all characters set as unordered (CI: 0.3640, RI: 0.7100). Values at nodes indicate Decay Index >1 and bootstrap >0.5. In this and subsequent figures the number following each species name indicates the mean value of the tip prior (in Mya). Points on nodes indicate the extents of node-based clade names: R, Russellosaurina; Te, Tethysaurinae; Y, Yaguarasaurinae. Lines on branches indicate the extents of branch-based clade names: A, Aigialosauridae; G, Globidensini; H, Halisaurinae; Ma, Mosasauroidea; Me, Mosasaurinae; Mi, Mosasaurini; N, Natantia; P, Plioplatecarpinae; Ty, Tylosaurinae. The lightning bolt symbol indicates the names that self-destruct under the topology provided.

**Figure 2:**
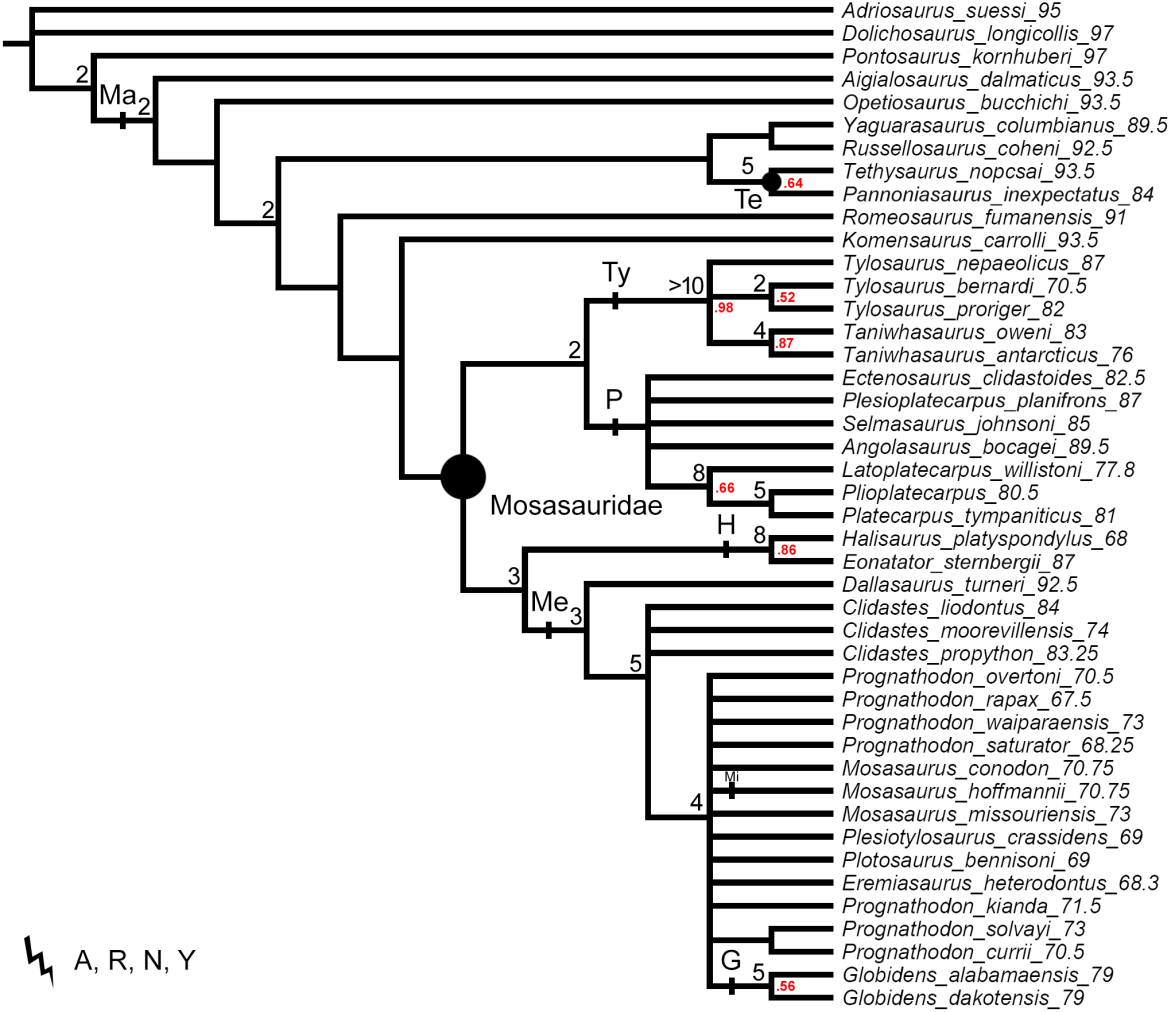
The strict consensus tree of 125 MPTs of length 465 inferred from unweighted parsimony analysis with a subset of multistate characters set as ordered (CI: 0.3484, RI: 0.7100). Values at nodes indicate Decay Index >1 and bootstrap >0.5. Points on nodes indicate the extents of node-based clade names: R, Russellosaurina; Te, Tethysaurinae; Y, Yaguarasaurinae. Lines on branches indicate the extents of branch-based clade names: A, Aigialosauridae; G, Globidensini; H, Halisaurinae; Ma, Mosasauroidea; Me, Mosasaurinae; Mi, Mosasaurini; N, Natantia; P, Plioplatecarpinae; Ty, Tylosaurinae. The lightning bolt symbol indicates the names that self-destruct under the topology provided.

The original data set of Simões et al. (2017) set all multistate characters as unordered. These settings were replicated for the first parsimony analysis to provide a better comparison of the Decay Index and bootstrap values behind the tree topologies resulting from ‘unweighted-unordered’ parsimony analysis (Fig. 1) and our ‘unweighted-ordered’ parsimony analysis (Fig. 2).

The decision to keep all characters unordered was not discussed neither justified, although it represents an implicit hypothesis on character-state transitions (see e.g., Wilkinson, 1992; Brazeau, 2011). We note that 19 among the multistate character statements in the character list of Simões et al. (2017) describe additive transformation series of nested states, and thus should be considered as ordered (the character statements: 1, 8, 10, 18, 20, 29, 30, 32, 37, 41, 53, 54, 55, 63, 72, 88, 96, 102, and 110). The setting of the above listed characters as unordered artificially excludes potential synapomorphies from the character sample, and may lead to the inference of spurious relationships (Brazeau, 2011). It is noteworthy that Simões et al. (2017) apparently recognized that some of these characters may be considered as ordered, but then left those characters as unordered (e.g., Simões et al., 2017,  Supplemental Information 1, see definition of state (1) of character 8, and comment on character statement 55).

The Decay Index and bootstrap values were calculated only in the two parsimony analyses with all characters having equal weight (‘unordered’ and ‘ordered’). The support values for the results inferred through the six runs of weighted parsimony (3 runs of ‘unordered’ settings for *K* = 3, 6, and 9; and 3 of ‘ordered’ settings for the same values of *K*) and the analyses with only one ‘dolichosaur’ included were not calculated. Rather, the inferred topologies resulting from these analyses are intended to visualize the effects of the use of different tree-search strategies (also, see ‘Discussion’ for comments on ‘Potential issues resulting from application of the Implied Weighting function’ and the ‘Outgroup selection’, that is particularly relevant when assessing the present results of parsimony analyses with only a single ‘dolichosaur’ included).

### Bayesian inference

Bayesian phylogenetic analysis integrating morphological and stratigraphic information was performed following the method discussed by Lee et al. (2014a), using implementations discussed by Lee et al. (2014b) and the Fossilized Birth–Death tree model sampling ancestors (FBDSA) introduced by Gavryushkina et al. (2014) and Gavryushkina et al. (2017). Bayesian inference analyses were performed in BEAST 2.4.4. (Drummond et al., 2012; Bouckaert et al., 2014), implemented with the packages for the analysis of morphological characters, using the model of Lewis (2001), and for sampling potential ancestors among the ingroup (Gavryushkina et al., 2014). The morphological matrix was the same as used in the parsimony analysis (see ‘Parsimony analyses’ above), with all characters set as unordered, to reproduce the settings used by Simões et al. (2017). Contrary to the outgroup used by previous analyses of mosasauroid affinities (‘composite’ outgroup and *Varanus*, see below and ‘Discussion’), Simões et al. (2017) added three early Late Cretaceous non-mosasauroid squamates, *Adriosaurus suessi* Seeley, 1881, *Dolichosaurus longicollis* Owen, 1850, and *Pontosaurus kornhuberi* Caldwell, 2006, and selected *A. suessi* as the root of the topologies. This outgroup selection is more realistic than the strategy followed in other recent analyses of Mosasauroidea, that use the extant and distantly-related *Varanus* (e.g., Palci, Caldwell & Papazzoni, 2013; Jiménez-Huidobro & Caldwell, 2016; Otero et al., 2017), since it assumes that the ancestral mosasauroid morphology is likely represented by the simplesiomorphies shared by penecontemporary semi-aquatic squamates close to the mosasauroid root. Furthermore, the use of Cenomanian squamates as mosasauroid outgroups does not violate uniform sampling rate required by the use of the FBDSA model. However, see the ‘Outgroup selection’ paragraph of ‘Discussion’ for further comments.

Since the character matrix did not include autapomorphies of the sampled taxa, the Lewis’s (2001) model was conditioned to variable characters only using the implementation included in BEAST 2.4.4. Stratigraphic information for the mosasauroid taxa was taken from the literature, and converted to geochronological ages. Stratigraphic data and age constraints for each terminal were obtained mainly from Polcyn et al. (2014) and integrated with information from the Paleobiology Database (http://paleobiodb.org/). The ages for *Romeosaurus fumanensis* and *Prognathodon kianda* were obtained from Palci, Caldwell & Papazzoni (2013) and Strganac et al. (2014), respectively. For the Bayesian analyses they performed, Simões et al. (2017) discussed the use of alternative distributions of the rate heterogeneity and rate frequency parameters, in particular, they suggested the use of a lognormal distribution instead of the more frequently used gamma distribution. In our analysis, rate variation across traits was modeled using the multi-gamma parameter (default model and unique implemented for the analysis of morphological data in BEAST 2). The rate variation across branches was modeled using the relaxed log-normal clock model, with the number of discrete rate categories that approximate the rate distribution set as *n* − 1 (with *n* the number of branches), the mean clock rate using default setting, and not setting to normalize the average rate. Particularly relevant for the taxonomic purposes of this study, the FBDSA tree model allows for testing whether one or more of the included taxa are sampled ancestors of one or more other included taxa, as it discriminates between cladogenetic and anagenetic patterns in macroevolution (Gavryushkina et al., 2014; Cau, 2017 and reference therein). We used two tree models included in the BEAST package: the Sampled Ancestor Fossilized Birth Death Skyline Model (Gavryushkina et al., 2014) and the FBDSA model (Gavryushkina et al., 2017). Convergence (stationarity) in numerical parameters among the different analyses was identified using Tracer (Rambaut & Drummond, 2009): the results showed broadly overlapping, non-trending traces across all replicate runs for every parameter, with effective sample sizes (ESS) of every parameter exceeding 100. Since all taxa included in the analysis are extinct, the rho parameter of Gavryushkina et al. (2014), which defines the probability to sample among extant taxa, was set as 0. The root age of the tree model was conservatively set as a uniform prior spanning between the age of the oldest ingroup taxa and 200 Mya (near the Triassic-Jurassic boundary: this age falls within the estimated range of the origin of the crown clade Squamata (Jones et al., 2013) though consistently pre-dates all known crown squamates (Conrad, 2008; Gauthier et al., 2012), and thus defines a time range that likely includes the age of the last common ancestor of all terminal taxa included). A first round of the analysis used four replicate runs of 10 million generations, with sampling every 1,000 generations, that were subsequently combined using LogCombiner 1.7.3 (included in the BEAST package). Then, we replicated the same analysis performing a single run of 40 million generations. In both analyses, burnin was set at 20%, and the Maximum Clade Credibility Tree (MCCT) used as framework for phyletic reconstruction. Convergence of parameters among the different runs was evaluated using Tracer. Exploration of the results of the alternative analyses produced identical topologies and did not indicate any significant differences in age inference. Given the overall overlap among the results of the alternative Bayesian analyses, for brevity, the following discussion refers to the analysis based on the single run of 40 million replications and using the FBDSA model. Although the MCCT is the topology with the maximum product of clade posterior probabilities, and is used for summarizing posterior distributions of trees (e.g., Lee et al., 2014b), it is necessary to remark that (1) not all relationships supported by the posterior distribution inferred are depicted in the MCCT, and (2) the most weakly-supported nodes included in the MCCT usually are recovered in small subsets of the posterior distribution. The half-compact consensus of the post-burnin topologies inferred (equivalent to a 50% majority rule consensus of the shortest trees, used in parsimony analyses) has been included for comparison with the MCCT (see Cau, 2017).

**Figure 3:**
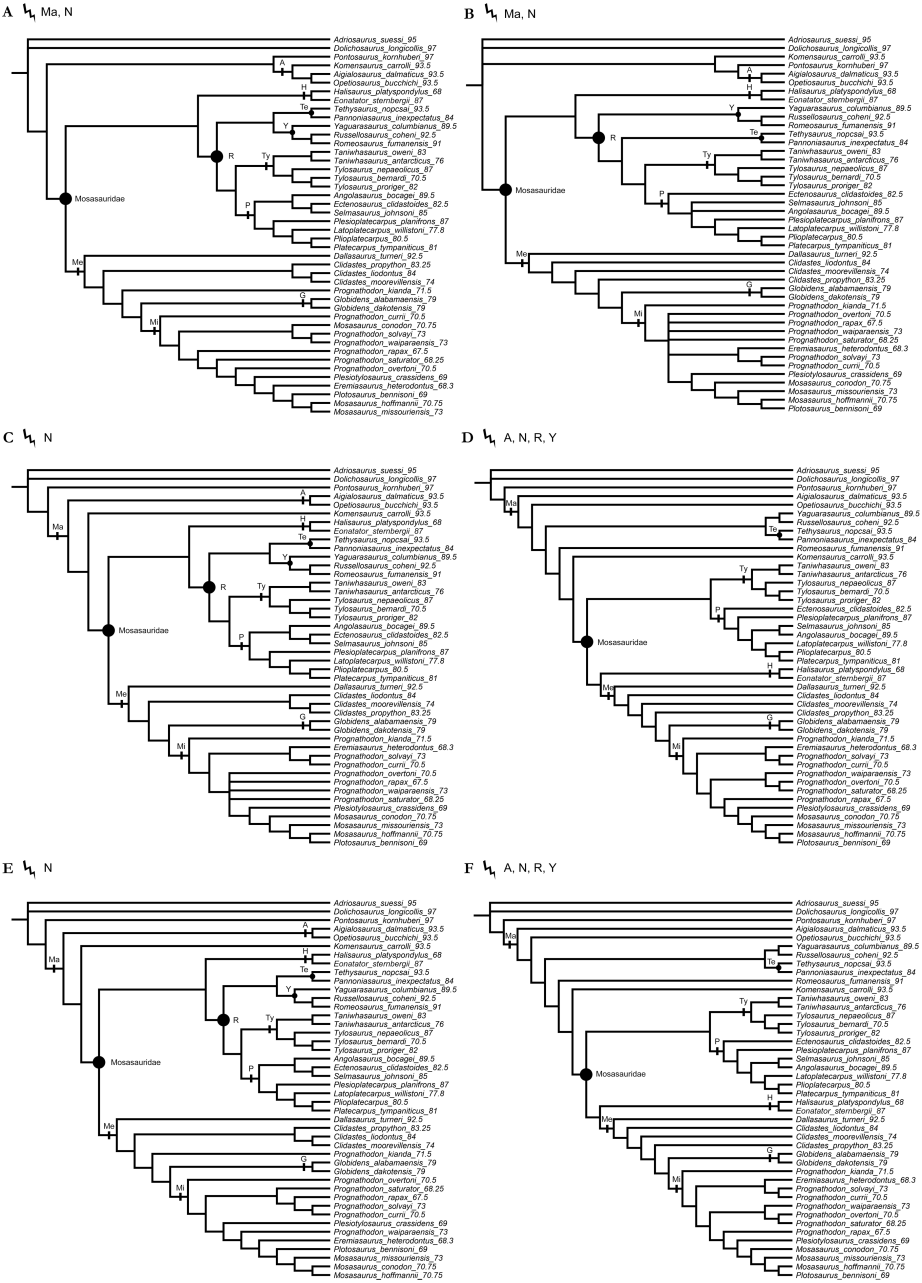
The strict consensus trees of the shortest topologies inferred from weighted parsimony analyses with all characters unordered (UO) and a subset of multistate characters set as ordered (O). (A) UO with *K* = 3 (1 MPT), (B) O with *K* = 3 (4 MPTs), (C) UO with *K* = 6 (2 MPTs), (D) O with *K* = 6 (1 MPT), (E) UO with *K* = 9 (1 MPT), (F) O with *K* = 9 (1 MPT). Points on nodes indicate the extents of node-based clade names: R, Russellosaurina; Te, Tethysaurinae; Y, Yaguarasaurinae. Lines on branches indicate the extents of branch-based clade names: A, Aigialosauridae; G, Globidensini; H, Halisaurinae; Ma, Mosasauroidea; Me, Mosasaurinae; Mi, Mosasaurini; N, Natantia; P, Plioplatecarpinae; Ty, Tylosaurinae. The lightning bolt symbol indicates the names that self-destruct under the topology provided.

**Figure 4:**
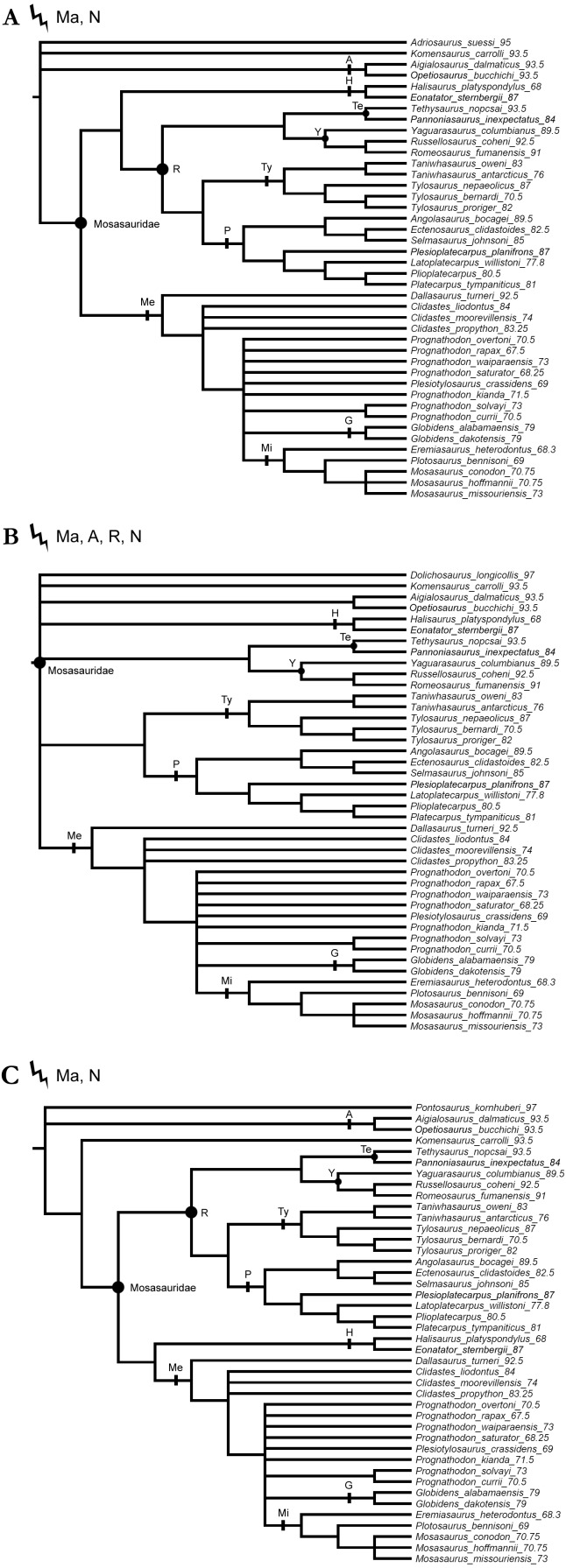
Strict consensus trees produced by the alternative tests using a single ‘dolichosaur’ taxon as outgroup. Trees rooted on (A) *Adriosaurus suessi* (40 MPTs), (B) *Dolichosaurus longicollis* (140 MPTs), and (C) *Pontosaurus kornhuberi* (20 MPTs) Points on nodes indicate the extents of node-based clade names: R, Russellosaurina; Te, Tethysaurinae; Y, Yaguarasaurinae. Lines on branches indicate the extents of branch-based clade names: A, Aigialosauridae; G, Globidensini; H, Halisaurinae; Ma, Mosasauroidea; Me, Mosasaurinae; Mi, Mosasaurini; N, Natantia; P, Plioplatecarpinae; Ty, Tylosaurinae. The lightning bolt symbol indicates the names that self-destruct under the topology provided.

## Results

**Figure 5:**
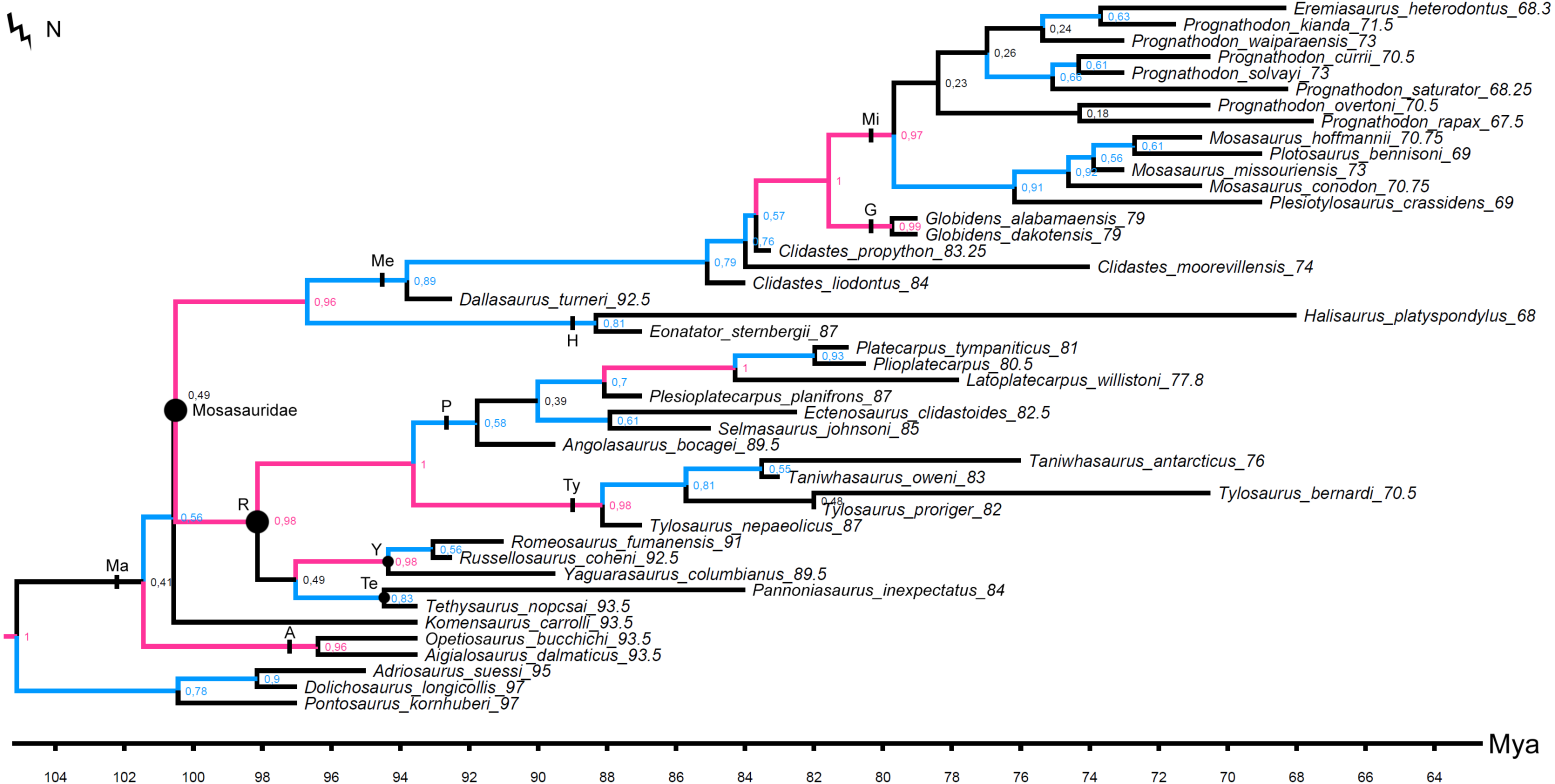
MCCT inferred by the Bayesian analysis. Branches colored according to posterior probability (*pp*) values: black, *pp* < 0.5; blue, 0.5 ≤ *pp* < 0.95; pink, *pp* ≥ 0.95. Points on nodes indicate the extents of node-based clade names: R, Russellosaurina; Te, Tethysaurinae; Y, Yaguarasaurinae. Lines on branches indicate the extents of branch-based clade names: A, Aigialosauridae; G, Globidensini; H, Halisaurinae; Ma, Mosasauroidea; Me, Mosasaurinae; Mi, Mosasaurini; N, Natantia; P, Plioplatecarpinae; Ty, Tylosaurinae. The lightning bolt symbol indicates the names that self-destruct under the topology provided.

**Figure 6:**
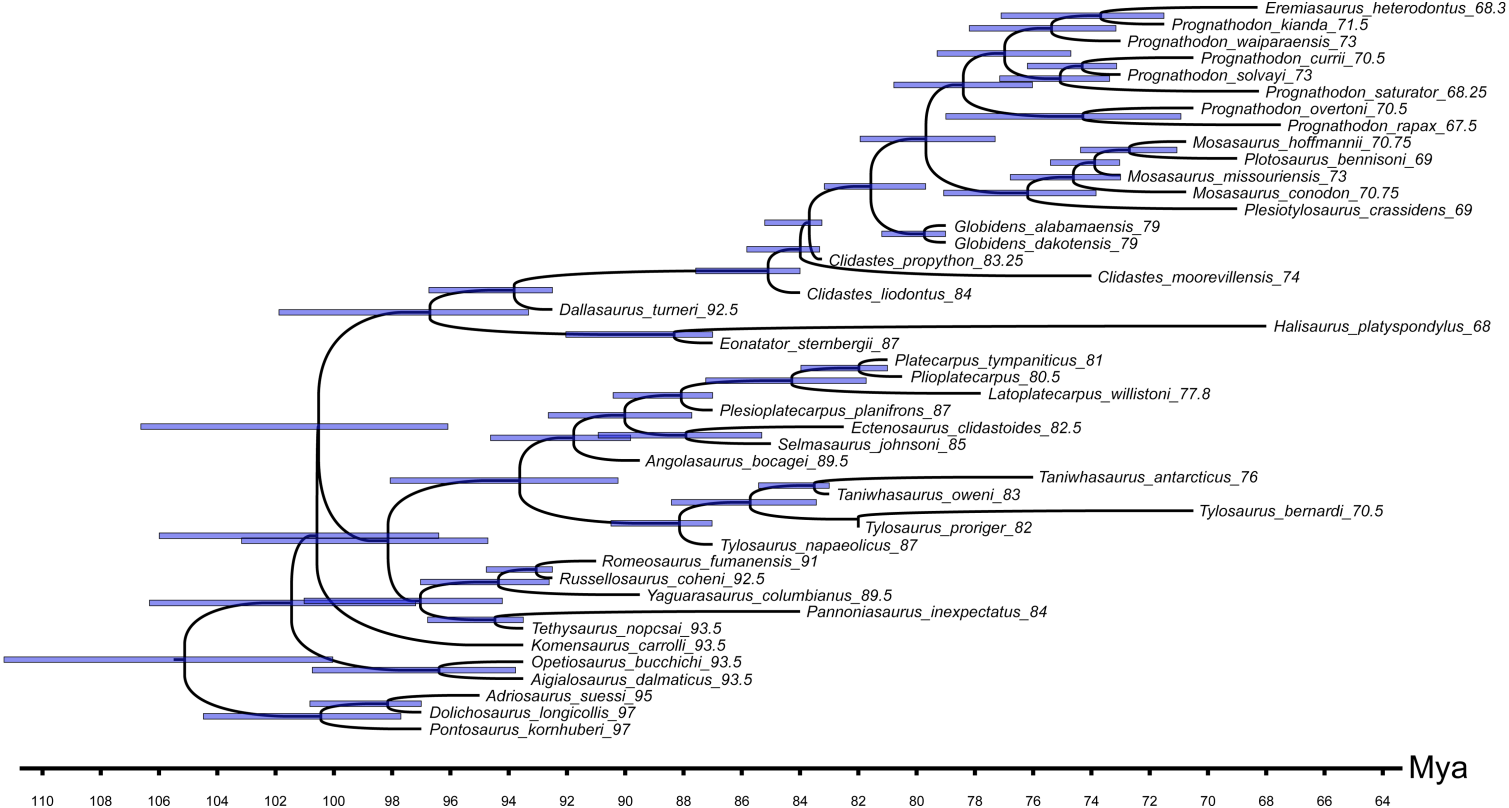
MCCT indicating the 95% confidence age range estimated for each node.

**Figure 7:**
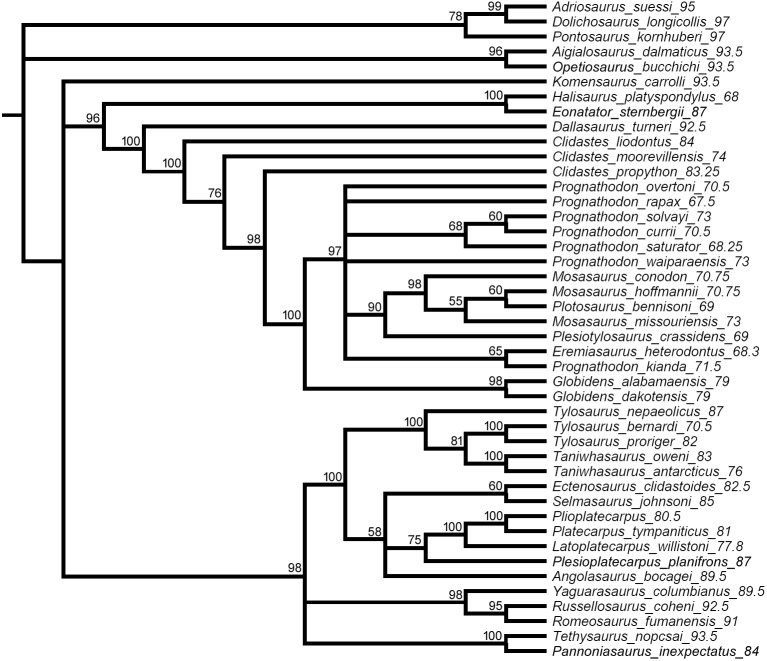
Half compact (majority rule) consensus of the topologies inferred among the post-burnin trees saved by the Bayesian analysis. Branch lengths not to scale. Numbers at nodes indicate % of sampled trees inferring those nodes.

All parsimony analyses (Figs. 1–4) and the Bayesian inference using the FBDSA model (Figs. 5–7) reconstruct most of the ‘traditionally’ recognized mosasaurid groups (Halisaurinae, Mosasaurinae, Plioplatecarpinae, Tethysaurinae, and Tylosaurinae) with the exception of Yaguarasaurinae which breaks down under the ‘unweighted-ordered’ parsimony analysis (Fig. 2) and two ‘weighted-ordered’ parsimony analyses (*K* = 6 and 9; Figs. 3D and 3F). However, the support behind the inferred nodes is generally poor with only a limited number of clades being strongly supported. The bootstrap and Decay Index (DI) values, which were calculated only in the ‘unweighted-unordered’ and ‘unweighted-ordered’ parsimony analyses using the full data set (i.e., when all three ‘dolichosaurs’ were included; Figs. 1 and 2), were highest for the clade Tylosaurinae (DI = 9 and >10, respectively; and bootstrap = 0.98) and the two species of the tylosaurine *Taniwhasaurus* (DI = 5 and 4; bootstrap = 0.80 and 0.87), and the clade Halisaurinae (DI = 7 and 8; bootstrap = 0.87 and 0.86). High values of DI were further calculated for the clade of advanced plioplatecarpines formed by *Latoplatecarpus willistoni*, *Platecarpus tympaniticus*, and *Plioplatecarpus* spp. (DI = 7 and 8, respectively), the clade of *P. tympaniticus* and *Plioplatecarpus* spp. (DI = 7 and 5), and the two species of the mosasaurine *Globidens* (DI = 8 and 5). However, the bootstrap values are <0.70 in all these groupings.

The Bayesian analysis strongly supports the monophyly of Tylosaurinae (posterior probability [*pp*] value = 0.98), the clade formed by *L. willistoni*, *P. tympaniticus*, and *Plioplatecarpus* spp. (*pp* = 1), and the monophyly of *Globidens* (*pp* = 0.99). However, the other groupings that were well supported by the parsimony analyses, have *pp* values below 0.95 (Halisaurinae: *pp* = 0.81; *Taniwhasaurus*: *pp* = 0.55). Interestingly, the Bayesian analysis strongly supports groupings that were not reconstructed by some parsimony analyses or only poorly supported, such as the Yaguarasaurinae (*pp* = 0.98) or the connection of Halisaurinae with Mosasaurinae (*pp* = 0.96). It also infers strong support for the grouping of advanced mosasaurines, including *Globidens*, the species attributed to *Prognathodon*, *Mosasaurus*, *Eremiasaurus*, *Plesiotylosaurus*, and *Plotosaurus* (*pp* = 1). In both parsimony analyses, for which the DI and bootstrap values were calculated, this grouping was reconstructed monophyletic as well but bootstrap was <0.50 (DI = 3 for ‘unweighted-unordered’ parsimony analysis and 4 for ‘unweighted-ordered’ parsimony analysis). Additionally, the Bayesian analysis strongly supports the grouping of tethysaurines, yaguarasaurines, plioplatecarpines, and tylosaurines (*pp* = 0.98) and a clade formed by plioplatecarpines and tylosaurines (*pp* = 1). In parsimony analyses, the former grouping was reconstructed only under the ‘unweighted-unordered’ settings but the DI was <2 and the bootstrap was <0.50. The latter grouping was inferred by both parsimony analyses but only the result of the ‘unweighted-ordered’ parsimony analysis showed the DI >1 (2). The bootstrap values were <0.50 in both cases. The Bayesian analysis also strongly supports the monophyly of *Aigialosaurus dalmaticus* and *Opetiosaurus bucchichi* (*pp* = 0.96), a grouping not inferred by the two parsimony analyses.

The Bayesian analysis inferred the age (and relative confidence interval) for each node (Figs. 5 and 6). The analysis estimated the divergence of the mosasauroids relative to the ‘dolichosaur’ outgroup during the Albian age (∼105 Mya), thus constraining the origin of the mosasauroid root during the last 6 million years of the Early Cretaceous. Focusing on the most robustly supported nodes in the MCCT (*pp* not less than 0.95), the mean age inferred for the *Aigialosaurus* + *Opetiosaurus* node is dated at ∼96 Mya (95% CI [94–100 Mya]), the mosasaurine-russellosaurinan divergence is dated at 100 Mya (95% CI [96–106.5 Mya]), the divergence of the Tylosaurinae and Plioplatecarpinae lineages is dated at ∼93.6 Mya (95% CI [90–98 Mya]), the origin of the last common ancestor of the included tylosaurine species is dated at 88 Mya (95% CI [87–90.5 Mya]), the lineage including *Latoplatecarpus willistoni*, *Plioplatecarpus* spp., and *Platecarpus tympanicus* originated at ∼84 Mya (95% CI [81.5–87 Mya]), the last common ancestor of mosasaurines and halisaurines is dated at ∼96.7 Mya (95% CI [93–102] Mya), the last common ancestor of Mosasaurini and Globidensini is dated at ∼81.6 Mya (95% CI [80–83 Mya]), the age of the last common ancestor of the two *Globidens* species included is dated at ∼80 Mya (95% CI [79–81 Mya]), and the last common ancestor of all mosasaurines closer to *M. hoffmannii* than *Globidens* is dated at ∼80 Mya (95% CI [77.3–82 Mya]).

### ‘Weak spots’ in the phylogeny of mosasauroids

The support and resolution is particularly poor near the base of the inferred trees. The ‘unweighted-unordered’ parsimony analysis shows an extensive basal polytomy and does not support the monophyly of mosasaurids exclusive of the ‘aigialosaurs’ (*Aigialosaurus dalmaticus* and *Opetiosaurus bucchichi*) and ‘dolichosaurs’ (Fig. 1). The ‘unweighted-ordered’ parsimony analysis groups halisaurines, mosasaurines, plioplatecarpines, tylosaurines, tethysaurines, and yaguarasaurines, but the support is weak (DI <2; bootstrap < 0.50). At the same time, it keeps tethysaurines outside ‘traditional’ mosasaurids (halisaurines, mosasaurines, plioplatecarpines, and tylosaurines) and does not support the monophyly of Yaguarasaurinae (Fig. 2). The Bayesian analysis, nevertheless, infers the monophyly of Mosasasauridae, consisting of monophyletic tethysaurines and yaguarasaurines, but the support is very low (*pp* = 0.49).

The weighted parsimony analyses and the analyses with a single ‘dolichosaur’ taxon included do not add much to the resolution either. Interestingly, however, there is a tendency, under some ‘ordered’ settings, to move the tethysaurines and yaguarasaurines (the latter being non-monophyletic) outside the ‘traditional’ mosasaurids when halisaurines are reconstructed as the sister taxon to mosasaurines (Figs. 2, 3D and 3F). There is also an apparent lack of resolution within the more advanced mosasaurines (the clade formed by *Globidens*, the species attributed to *Prognathodon*, *Mosasaurus*, *Eremiasaurus*, *Plesiotylosaurus*, and *Plotosaurus*), which are, nevertheless, inferred monophyletic by all analyses (Figs. 1–7; see also above for the support of this grouping). The most striking is the non-monophyly of *Prognathodon* (inferred also by other authors; e.g., Leblanc, Caldwell & Bardet, 2012; Simões et al., 2017). Some analyses unite certain taxa assigned to *Prognathodon*, but only the monophyly of *P. solvayi* and *P. currii* is reconstructed consistently (Figs. 1–7; except for Fig. 3A), though still poorly supported (DI < 2; bootstrap <0.50; *pp* = 0.61).

Further, the monophyly of *Clidastes* is supported only by ‘weighted-unordered’ parsimony analyses; regardless of the value of *K* (Figs. 3A, 3C, 3E). All other analyses, including the Bayesian inference, keep *Clidastes* paraphyletic relative to other mosasaurines.

### Phylogenetic nomenclature

Inferred phylogenetic relationships are further discussed within the context of mosasauroid systematics and used as the primary basis for nomenclatural revision of the main mosasauroid clades.

The recommended phylogenetic definitions applied for the taxon names follow the *International Code of Phylogenetic Nomenclature*, or *PhyloCode*, hereafter *ICPN* (Cantino & De Queiroz, 2010). They are summarized in Table 1. Likewise, the taxon names are attributed to the authors that introduced them (following the *ICPN*: Art. 9.8, Note 9.8A.2), and not according to the Principle of Coordination (ICZN, 1999: Art. 36). This approach is preferred due to its more transparent account of the original literature.

**Table 1:** Recommended phylogenetic definitions applied to mosasauroid taxon names.

Clade name	Internal specifier(s)	External specifier(s)	Type of phylogenetic definition	Authorship
Mosasauroidea	*Mosasaurus hoffmannii*, *Aigialosaurus dalmaticus*	*Dolichosaurus longicollis*, *Adriosaurus suessi*, *Pontosaurus lesinensis*	Branch-based	New
Aigialosauridae	*Aigialosaurus dalmaticus*, *Opetiosaurus bucchichi*	*Dolichosaurus longicollis*, *Adriosaurus suessi*, *Pontosaurus lesinensis*, Mosasauridae = (*Mosasaurus hoffmannii*, *Halisaurus platyspondylus*, *Tylosaurus proriger*)	Branch-based	New
Mosasauridae	*Mosasaurus hoffmannii*, *Halisaurus platyspondylus*, *Tylosaurus proriger*		Node-based	Madzia & Conrad (in press)
Halisaurinae	*Halisaurus platyspondylus*	*Mosasaurus hoffmannii*, *Tylosaurus proriger*, *Tethysaurus nopcsai*, *Yaguarasaurus columbianus*	Branch-based	New
Natantia	*Mosasaurus hoffmannii*, *Tylosaurus proriger*, *Plioplatecarpus marshii*	*Halisaurus platyspondylus*	Branch-based	Conrad (2008)
Mosasaurinae	*Mosasaurus hoffmannii*	*Tylosaurus proriger*, *Plioplatecarpus marshii*, *Halisaurus platyspondylus*, *Tethysaurus nopcsai*, *Yaguarasaurus columbianus*	Branch-based	New
Mosasaurini	*Mosasaurus hoffmannii*	*Globidens alabamaensis*	Branch-based	New
Globidensini	*Globidens alabamaensis*	*Mosasaurus hoffmannii*	Branch-based	New
Russellosaurina	*Russellosaurus coheni*, *Tylosaurus proriger*, *Plioplatecarpus marshii*	*Mosasaurus hoffmannii*	Node-based	New
Tethysaurinae	*Tethysaurus nopcsai*, *Pannoniasaurus inexpectatus*	*Halisaurus platyspondylus*, *Mosasaurus hoffmannii*, *Tylosaurus proriger*, *Plioplatecarpus marshii*, *Yaguarasaurus columbianus*	Node-based	New
Yaguarasaurinae	*Yaguarasaurus columbianus*, *Russellosaurus coheni*, *Romeosaurus fumanensis*	*Tethysaurus nopcsai*, *Halisaurus platyspondylus*, *Tylosaurus proriger*, *Plioplatecarpus marshii*, *Mosasaurus hoffmannii*	Node-based	New
Plioplatecarpinae	*Plioplatecarpus marshii*	*Mosasaurus hoffmannii*, *Tylosaurus proriger*, *Tethysaurus nopcsai*, *Yaguarasaurus columbianus*	Branch-based	New
Tylosaurinae	*Tylosaurus proriger*	*Plioplatecarpus marshii*, *Mosasaurus hoffmannii*	Branch-based	Conrad (2008)

Even though the majority of the preferred phylogenetic definitions is labeled as ‘new’ (see Table 1), most of them merely represent modified versions of the definitions proposed by other authors. We attempted to provide only the necessary changes to maintain the traditional meaning of the clade names and to maximize their stability given the inferred ‘weak spots’ in the mosasauroid phylogenetic tree.

### Mosasauroidea Camp, 1923

#### Preferred phylogenetic definition

The most inclusive clade containing *Mosasaurus hoffmannii* Mantell, 1829 and *Aigialosaurus dalmaticus* Kramberger, 1892, but not *Dolichosaurus longicollis* Owen, 1850, *Adriosaurus suessi* Seeley, 1881, or *Pontosaurus lesinensis* Kornhuber, 1873. This definition is branch-based.

#### Remarks

Mosasauroidea traditionally includes mosasaurids and ‘aigialosaurs’ (e.g., Bell, 1997; Bell & Polcyn, 2005; Conrad, 2008). Proper delimitation of the extent of the name Mosasauroidea, however, requires adequate knowledge of the early evolution of Mosasauria and reappraisal of the phylogenetic positions of potential non-mosasauroid mosasaurs (e.g., the species belonging to *Adriosaurus*, *Pontosaurus*, *Dolichosaurus*). These taxa, or their subset, have been hypothesized to be either more closely related to snakes (see e.g., Palci & Caldwell, 2007; Caldwell & Palci, 2010; Palci & Caldwell, 2010) or to mosasaurids (e.g., Reeder et al., 2015). Considering that (1) the ‘dolichosaurs’ are traditionally regarded as non-mosasauroids, and (2) ‘aigialosaurs’ and mosasaurids are frequently inferred more closely related to each other than either is to the ‘dolichosaurs’, we propose a new definition that seems to adhere to the traditional use of Mosasauroidea (i.e., ‘aigialosaurs’ plus mosasaurids, but not ‘dolichosaurs’) and reflects the uncertainties surrounding the phylogenetic placements of near-mosasaurids and early mosasaurids as inferred, among others, in the present study (see Figs. 1–7).

### Aigialosauridae Kramberger, 1892

#### Preferred phylogenetic definition

The most inclusive clade containing *Aigialosaurus dalmaticus* Kramberger, 1892 and *Opetiosaurus bucchichi* Kornhuber, 1901 but not *Dolichosaurus longicollis* Owen, 1850, *Adriosaurus suessi* Seeley, 1881, *Pontosaurus lesinensis* Kornhuber, 1873, or the clade originating with the most recent common ancestor of *Halisaurus platyspondylus* Marsh, 1869, *Mosasaurus hoffmannii* Mantell, 1829, and *Tylosaurus proriger* (Cope, 1869). This definition is branch-based.

#### Remarks

Aigialosauridae has a long and problematic history. The last thorough review of the interrelationships of early Mosasauria, i.e., those species associated with the evolutionary transition to aquatic lifestyle, was published by Dutchak (2005) who concluded that “redescriptions of the key taxa (*Aigialosaurus dalmaticus*, *Opetiosaurus bucchichi* and ‘the Trieste aigialosaur’) are essential to further investigations into re-testing the most recent hypotheses” (p. 228). Although *A. dalmaticus* and *O. bucchichi* have since been redescribed (Dutchak & Caldwell, 2006; Dutchak & Caldwell, 2009; respectively), and ‘the Trieste aigialosaur’ was assessed and given the name *Komensaurus carrolli* (Caldwell & Palci, 2007), the status of Aigialosauridae did not change. Indeed, Dutchak & Caldwell (2009) argued that *O. bucchichi* should be assigned to *Aigialosaurus* (as *A. bucchichi*), suggesting close relationships of the two taxa. Still, their analysis does not necessarily support this conclusion (see Dutchak & Caldwell, 2009: Fig. 4).

While it is certainly possible that *A. dalmaticus* and *O. bucchichi* are more closely related to one another than either is to other mosasauroids, such a result is currently not strongly supported statistically. The ‘full’ parsimony analyses (with all ‘dolichosaurs’ included and *A. suessi* selected as outgroup) reconstruct the taxa in a basal polytomy with other mosasauroid subclades (Fig. 1) or as successively more closely related to mosasaurids, with *A. dalmaticus* being the more basal of the two (Fig. 2). The Bayesian inference, majority of the weighted parsimony analyses (except for Figs. 3D and 3F), and parsimony analyses using different ‘dolichosaurs’ as outgroups, nevertheless, reconstruct a clade formed by both these species (Figs. 3–5), though their position on the mosasauroid tree is unstable.

Considering the problematic nature of mosasauroid origins, we admit that Aigialosauridae might be of use in the future. In this case, however, we strongly encourage using a complex self-destructive phylogenetic definition to reflect the history of the name as well as its unstable contents (see *ICPN*: Art. 11.9). The self-destructive branch-based definition that is proposed here keeps Aigialosauridae in use only if *A. dalmaticus* and *O. bucchichi* are more closely related to each other than either is to ‘dolichosaurs’ or Mosasauridae *sensu* Madzia & Conrad (in press). Also, it does not allow the use of the name in the cases when *A. dalmaticus* and *O. bucchichi* are reconstructed within Mosasauridae.

### Mosasauridae Gervais, 1853

#### Preferred phylogenetic definition

The least inclusive clade containing *Mosasaurus hoffmannii* Mantell, 1829, *Halisaurus platyspondylus* Marsh, 1869, and *Tylosaurus proriger*. This definition is node-based.

#### Remarks

The history of the name Mosasauridae, its approximate synonyms, and its application were discussed by Madzia & Conrad (in press) who also provided the phylogenetic definition for the clade name as will be recognized by the *ICPN*.

The Bayesian analysis and parsimony analyses using different ‘dolichosaurs’ as the outgroup maintain the monophyly of mosasaurines, plioplatecarpines, tylosaurines, tethysaurines, yaguarasaurines, and the two halisaurine species. The ‘unweighted-ordered’ parsimony analysis, however, reconstructs tethysaurines and yaguarasaurines outside Mosasauridae, with *Romeosaurus* being inferred as the sister taxon to *Komensaurus carrolli* + mosasaurids, outside tethysaurines + a clade formed by *Yaguarasaurus* and *Russellosaurus* (Fig. 2). Thus, it makes Yaguarasaurinae polyphyletic.

The mutual relationships of particular mosasaurid clades are unsettled and highly dependent on the tree-search strategies used (Figs. 1–7). Still, even though the hypotheses of mosasaurid interrelationships are differing, the definition proposed by Madzia & Conrad (in press) does not require modifications. It covers all ‘traditional’ mosasaurid taxa, including the plioplatecarpines. Though not represented in the phylogenetic definition, *Plioplatecarpus* and its kin are kept within Mosasauridae under all inferred topologies.

### Halisaurinae Bardet et al., 2005

#### Preferred phylogenetic definition

The most inclusive clade containing *Halisaurus platyspondylus* Marsh, 1869, but not *Mosasaurus hoffmannii* Mantell, 1829, *Tylosaurus proriger* (Cope, 1869), *Tethysaurus nopcsai* Bardet, Suberbiola & Jalil, 2003, or *Yaguarasaurus columbianus* Páramo, 1994. This definition is branch-based.

#### Remarks

Bardet et al. (2005) defined Halisaurinae as “Mosasauridae more closely related to *Halisaurus* than to *Mosasaurus*” (p. 464). Later, Conrad (2008) used equivalent branch-based definition with type species as specifiers: “All taxa sharing a more recent common ancestor with *Halisaurus platyspondylus* than *Mosasaurus hoffmannii*” (p. 127). Because the position of the species for which the name Halisaurinae was proposed is not very stable within Mosasauroidea (see the results of the present analysis and the Natantia paragraph below), we consider the proposed branch-based definition including additional external specifiers, representing other inferred clades, to be the most appropriate one.

Nevertheless, the current data set is not fully suitable for testing the phylogenetic position of Halisaurinae within Mosasauridae as the clade is represented by only two taxa (*H. platyspondylus* and *Eonatator sternbergii*).

### Natantia Owen, 1851

#### Preferred phylogenetic definition

The most inclusive clade containing *Mosasaurus hoffmannii* Mantell, 1829, *Tylosaurus proriger* (Cope, 1869), and *Plioplatecarpus marshii* Dollo, 1882, but not *Halisaurus platyspondylus* Marsh, 1869. This definition is branch-based.

#### Remarks

Bell (1997) resurrected the name Natantia from the mid-nineteenth century (Owen, 1851). It was used to unite Bell’s (1997) ‘Russellosaurinae’ (see the Russellosaurina paragraph) and Mosasaurinae, exclusive of the *Halisaurus* species and the ‘aigialosaurs.’ Conrad (2008: 128) proposed the following branch-based definition: “All taxa sharing a more recent common ancestor with *Mosasaurus hoffmanni*, *Tylosaurus proriger*, and *Plioplatecarpus marshi* than with *Halisaurus platyspondylus*”. When applied on some recent phylogenetic hypotheses based on the data set initially published by Bell & Polcyn (2005), that infer halisaurines to be nested within the smallest clade containing *Mosasaurus*, *Tylosaurus*, and *Plioplatecarpus*, Natantia self-destructs.

Our analyses do not support the concept of Natantia either (Figs. 1–7). In the ‘unweighted-ordered’ parsimony analysis (Fig. 2), some weighted parsimony analyses (Figs. 3D and 3F), parsimony analysis with *Pontosaurus* as the outgroup (Fig. 4C), and Bayesian analysis (Fig. 5), halisaurines form the sister taxon to mosasaurines. When *Adriosaurus* is used as outgroup, and other ‘dolichosaurs’ are excluded, and under some weighted parsimony analyses, halisaurines are more closely related to the clade formed by tethysaurines, yaguarasaurines, tylosaurines, and plioplatecarpines than to mosasaurines (Figs. 3A– 3C, 3E and 4A).

It is worth noting that Boas (1880) used the name Natantia for a subgroup of decapod crustaceans. Although Owen’s (1851) Natantia was published earlier, the priority issue is problematic. The ICZN (1999) does not govern the names above the family group, and Natantia, approximately corresponding to the concept of Owen (1851), had not been in use until Bell (1997). Similarly, the use of Boas (1880) is outdated (WoRMS, 2015), though it was of importance in the past (see, for example, the discussion in Felgenhauser & Abele, 1983).

We refrain from providing a lengthy discussion of the nomenclatural issue or a solution to it but since the name Natantia Owen (1851) was published earlier, we provisionally keep it as the name for the potential grouping as discussed above.

### Mosasaurinae Williston, 1897

#### Preferred phylogenetic definition

The most inclusive clade containing *Mosasaurus hoffmannii* (Mantell, 1829), but not *Tylosaurus proriger* (Cope, 1869), *Plioplatecarpus marshii* Dollo, 1882, *Halisaurus platyspondylus* Marsh, 1869, *Tethysaurus nopcsai* Bardet, Suberbiola & Jalil, 2003, or *Yaguarasaurus columbianus* Páramo, 1994. This definition is branch-based.

#### Remarks

Mosasaurinae is traditionally considered to represent a species-rich clade with substantial morphological and ecological diversity (e.g., Bell, 1997; Bell & Polcyn, 2005; Bardet et al., 2015).

The first published phylogenetic definition is the following: “All taxa sharing a more recent common ancestor with *Mosasaurus hoffmanni* than with *Tylosaurus proriger* or *Plioplatecarpus marshi*” (Conrad, 2008: 128). This branch-based definition keeps the traditional contents of Mosasaurinae intact when applied to the majority of recent analyses. We added additional external specifiers, *Halisaurus platyspondylus*, *Tethysaurus nopcsai*, and *Yaguarasaurus columbianus*, to reflect the traditional contents of Mosasaurinae and the inferred overall instability in the mosasaurid interrelationships. The monophyly of mosasaurines, however, is inferred by all our analyses (Figs. 1–7).

### Mosasaurini Russell, 1967

#### Preferred phylogenetic definition

The most inclusive clade containing *Mosasaurus hoffmannii* Mantell, 1829, but not *Globidens alabamaensis* Gilmore, 1912. This definition is branch-based.

#### Remarks

Bell (1997: 322) abandoned Mosasaurini on the basis of the supposed paraphyly of *Mosasaurus* and “expanded [Plotosaurini] to include basic taxa previously referred to *Mosasaurus*”. Both taxon names, Mosasaurini and Plotosaurini, were introduced in the same publication (Russell, 1967). However, it seems that the former has gained more attention (e.g., Leblanc, Caldwell & Bardet, 2012; Fanti, Cau & Negri, 2014). Leblanc, Caldwell & Bardet (2012: 101) argued to replace Plotosaurini with Mosasaurini which they used for “the group consisting of (*Eremiasaurus* (*Mosasaurus* + *Plotosaurus*))”. Although the close connection of these taxa is generally supported by recent phylogenetic studies (e.g., Grigoriev, 2013; Palci, Caldwell & Papazzoni, 2013; Fanti, Cau & Negri, 2014; Jiménez-Huidobro & Caldwell, 2016), analyses using multiple tree-search strategies show conflicting results (Simões et al., 2017). The grouping is maintained in the ‘unweighted-unordered’ parsimony analysis, under one ‘weighted-unordered’ parsimony analysis (Fig. 3E), and when only one of the ‘dolichosaur’ taxa is included (Fig. 4). Still, ‘unweighted-ordered’ parsimony, other weighted parsimony analyses, and the Bayesian inference fail to support such topology.

### Globidensini Russell, 1967

#### Preferred phylogenetic definition

The most inclusive clade containing *Globidens alabamaensis* (Gilmore, 1912), but not *Mosasaurus hoffmannii* Mantell, 1829. This definition is branch-based.

#### Remarks

Bell (1997) used Russell’s (1967) Globidensini to unite *Globidens*, *Prognathodon*, and *Plesiotylosaurus*. Although such close connection of these taxa is not necessarily supported by current studies (e.g., Palci, Caldwell & Papazzoni, 2013; Fanti, Cau & Negri, 2014; Jiménez-Huidobro & Caldwell, 2016), there is indeed a tendency to keep them together under the name Globidensini (e.g., Schulp et al., 2008; Leblanc, Caldwell & Bardet, 2012). Nevertheless, forcing *Prognathodon solvayi*, the type species of *Prognathodon*, to be a globidensin (by selecting it as an internal specifier), would be potentially ineffective considering the likely para- or even polyphyletic nature of the taxa attributed to *Prognathodon*.

All our analyses fail to reconstruct Globidensini with more than only the two species of *Globidens* included (Figs. 1–7). Nevertheless, the clade name may still be useful for discussions related to mosasaurid ecology (due to the specialized dentition of *Globidens* and *Carinodens*, its potential close relative (Schulp, Jagt & Fonken, 2004)).

### Russellosaurina Polcyn & Bell, 2005

#### Preferred phylogenetic definition

The least inclusive clade containing *Russellosaurus coheni* Polcyn & Bell, 2005, *Tylosaurus proriger* (Cope, 1869), and *Plioplatecarpus marshii* Dollo, 1882, but not *Mosasaurus hoffmannii* Mantell, 1829. This definition is node-based.

#### Remarks

Due to its problematic history, the name Russellosaurina is discussed here in detail. In his PhD thesis, Bell (1993) proposed a new name, Russellosaurinae, to link tylosaurines and plioplatecarpines together, and provided the following node-based definition: “The most recent common ancestor of *Tylosaurus*, *Ectenosaurus* and *Plioplatecarpus* and all of its descendants” (p. 183). He noted that Russellosaurinae consists of “*Tylosaurus* and Plioplatecarpini” (p. viii), which matched his definition. Bell’s PhD thesis was published four years later (Bell, 1997). Until that time, ‘Russellosaurinae’ was in use in an informal sense as a node-based name for a clade consisting of ‘tylosaurines’ and ‘plioplatecarpines’ (Caldwell, 1996). Because the paper by Bell (1997) was originally intended to simply be the published version of his PhD thesis, Bell (1997) again introduced ‘Russellosaurinae’ as a new taxon name. However, its extent seems to be different as the name was introduced “in anticipation of formally designating the taxon and describing a new taxon, *Russellosaurus*, from new Turonian material from Texas” (p. 322). Although there was no explicit information about how closely related *Russellosaurus* was to ‘russellosaurines’ (*sensu* Bell, 1993), and in the ‘Summary’ paragraph of Bell (1997: 324) ‘Russellosaurinae’ is again listed as consisting of “*Tylosaurus* and Plioplatecarpini” only, it is clear that Bell (1997) intended to anchor ‘Russellosaurinae’ on the taxon *Russellosaurus*. Until Polcyn & Bell (2005), where ‘Russellosaurinae’ was officially replaced with Russellosaurina, authors used the name in the traditional informal way, and always as a node-based name for a clade containing *Tylosaurus* and Plioplatecarpini (Christiansen & Bonde, 2002) or Plioplatecarpinae (Bardet et al., 2005); the latter two names referring to the same content.

Polcyn & Bell (2005) introduced the name Russellosaurina “to give identity to the monophyletic grouping of Tylosaurinae plus Plioplatecarpinae and closely related forms” (Polcyn & Bell, 2005: 323). What the “closely related forms” are is clear from the ‘Systematic palaeontology’ paragraph (p. 322), according to which the only non-mosasaurine mosasaurid taxa listed there as Russellosaurina are “[t]he subfamilies Tylosasaurinae [sic] and Plioplatecarpinae and their sister-clade containing the genera *Tethysaurus*, *Russellosaurus* and *Yaguarasaurus*”. Unfortunately, the composition of Russellosaurina is not that transparent in other parts of that paper. According to the abstract, Russellosaurina “includes Plioplatecarpinae, Tylosaurinae, their [most recent] common ancestor and all [of its] descendants” (p. 321), and according to the phylogenetic definition, Russellosaurina consists of “[a]ll mosasaurs more closely related to Tylosaurinae and Plioplatecarpinae, the genus *Tethysaurus*, their common ancestor and all descendants than to Mosasaurinae” (p. 322). This definition is clearly branch-based with “Tylosaurinae and Plioplatecarpinae, the genus *Tethysaurus*, their common ancestor and all descendants” being a node-based clade and an internal specifier of the definition. This wording is therefore inconsistent with all previously cited statements.

When Polcyn & Bell (2005) established the name, they gave it the rank of ‘parafamily,’ a term introduced by Olshevsky (1991) for ‘paraphyletic family’ (the prefix ‘para-’ indicates ‘paraphyly’), and not recognized by the *ICZN*. Therefore, it is of the same level as ‘family’. However, the suffix ‘-ina’ typically indicates a subtribe in zoological nomenclature, so when assigning the name Russellosaurina a rank, the taxon should be contained within a tribe and a subfamily. Here, Russellosaurina is considered an unranked clade name with the node-based definition provided above. In our definition, *M. hoffmannii* is used as a qualifying clause (*ICPN*: Art. 11.9). The suggested compilation is preferred for various reasons. First, it should “[supersede] previous references to ‘Russellosaurinae”’ (Polcyn & Bell, 2005: 323), thus applying to the clade originating with the most recent common ancestor of Tylosaurinae, Plioplatecarpinae, and *R. coheni*. Further, Russellosaurina has always been understood as a node-based name. Although Conrad (2008) “tentatively” followed the original branch-based definition, he simultaneously noted that “the definition Polcyn & Bell (2005) intended for Russellosaurina is frustratingly ambiguous” (Conrad, 2008: 129). Since *R. coheni* was omitted from the specifiers, the original definition violated the *ICPN* (Art. 11.7).

According to the new definition, Russellosaurina contains the species *R. coheni*, *Y. columbianus*, *T. nopcsai*, the clade Plioplatecarpinae, and the clade Tylosaurinae (as inferred, e.g., in Bell & Polcyn, 2005; Dutchak & Caldwell, 2006; Cuthbertson et al., 2007). It may also contain Halisaurinae as reconstructed in Caldwell & Palci (2007), or self-destruct under the hypothesis from Bardet et al. (2005). Russellosaurina may also contain only Plioplatecarpinae and Tylosaurinae, if *R. coheni* and *Y. columbianus* are basal members of Plioplatecarpinae as it was suggested by Polcyn & Bell (2005: 332) and inferred in Dutchak & Caldwell (2009: Fig. 5). Russellosaurina self-destructs if *R. coheni*, *Y. columbianus* and *T. nopcsai* form the sister taxon to the least inclusive clade including *M. hoffmannii* and *T. proriger* as reconstructed in Dutchak & Caldwell (2009: Fig. 4).

The ‘unweighted-unordered’ parsimony analysis (Fig. 1), some weighted parsimony analyses (Figs. 3A–3C and 3E), parsimony analyses with *Adriosaurus* and *Pontosaurus* used as outgroups (Figs. 4A and 4C), and Bayesian analysis (Fig. 5) support Russellosaurina. Under all other topologies, Russellosaurina self-destructs (Figs. 2, 3D, 3F and 4B).

### Tethysaurinae Makádi, Caldwell & Ösi, 2012

#### Preferred phylogenetic definition

The least inclusive clade containing *Tethysaurus nopcsai* Bardet, Suberbiola & Jalil, 2003 and *Pannoniasaurus inexpectatus* Makádi, Caldwell & Ösi, 2012, but not *Halisaurus platyspondylus* Marsh, 1869, *Mosasaurus hoffmannii* (Mantell, 1829), *Tylosaurus proriger* (Cope, 1869), *Plioplatecarpus marshii* Dollo, 1882, or *Yaguarasaurus columbianus* Páramo, 1994. This definition is node-based.

#### Remarks

Makádi, Caldwell & Ösi (2012) introduced the name Tethysaurinae for “[t]he most recent common ancestor of *Pannoniasaurus inexpectatus* and *Russellosaurus coheni* Polcyn & Bell, 2005 […] and all its descendants”. Following the results of their phylogenetic analysis, the clade Tethysaurinae was formed by *P. inexpectatus*, *R. coheni*, *Tethysaurus nopcsai*, and *Yaguarasaurus columbianus*. However, by omitting *T. nopcsai* from the internal specifiers, the phylogenetic definition violates the *ICPN* (Art. 11.7). Later, Palci, Caldwell & Papazzoni (2013) introduced the name Yaguarasaurinae and defined it as “[t]he most recent common ancestor of *Romeosaurus*, gen. nov., *Russellosaurus*, and *Yaguarasaurus*, and all of its descendants”. Tethysaurinae was kept only for *Pannoniasaurus* and *Tethysaurus*, that formed the sister clade to the Yaguarasaurinae (see below for comments on this name).

We follow the node-based concept of Tethysaurinae as delimited by Palci, Caldwell & Papazzoni (2013) but considering the unstable position of the two tethysaurines on the mosasauroid tree (see Figs. 1–7), we added five external specifiers to maintain the ‘traditional’ contents.

All our analyses reconstruct monophyletic tethysaurines (Figs. 1–7).

### Yaguarasaurinae Palci, Caldwell & Papazzoni, 2013

#### Preferred phylogenetic definition

The least inclusive clade containing *Yaguarasaurus columbianus* Páramo, 1994, *Russellosaurus coheni* Polcyn & Bell, 2005, and *Romeosaurus fumanensis* Palci, Caldwell & Papazzoni, 2013, but not *Tethysaurus nopcsai* Bardet, Suberbiola & Jalil, 2003, *Halisaurus platyspondylus* Marsh, 1869, *Tylosaurus proriger* (Cope, 1869), *Plioplatecarpus marshii* Dollo, 1882, or *Mosasaurus hoffmannii* Mantell, 1829. This definition is node-based.

#### Remarks

As noted above, Yaguarasaurinae was introduced by Palci, Caldwell & Papazzoni (2013) who defined it as “[t]he most recent common ancestor of *Romeosaurus*, gen. nov., *Russellosaurus*, and *Yaguarasaurus*, and all of its descendants”. We follow such definition but considering the weak support for the connection of Yaguarasaurinae and Tethysaurinae (Figs. 1, 2, 5 and 7), we added five external specifiers to prevent the name to cover an unintended clade.

The Bayesian analysis and majority of the parsimony analyses support the monophyly of the yaguarasaurines as delimited by Palci, Caldwell & Papazzoni (2013). Only under the topology resulting from the ‘unweighted-ordered’ parsimony analysis and two ‘weighted-ordered’ parsimony analyses Yaguarasaurinae self-destructs (Figs. 2, 3D and 3F).

### Plioplatecarpinae Dollo, 1884

#### Preferred phylogenetic definition

The most inclusive clade containing *Plioplatecarpus marshii* Dollo, 1882, but not *Mosasaurus hoffmannii* Mantell, 1829, *Tylosaurus proriger* (Cope, 1869), *Tethysaurus nopcsai* Bardet, Suberbiola & Jalil, 2003, or *Yaguarasaurus columbianus* Páramo, 1994. This definition is branch-based.

#### Remarks

Conrad (2008: 130) defined Plioplatecarpinae as “[a]ll taxa sharing a more recent common ancestor with *Plioplatecarpus marshi*[*i* ] than with *Tylosaurus proriger* or *Mosasaurus hoffmannii*”. Such definition matches the published hypotheses; Plioplatecarpinae as sister taxon to Tylosaurinae or to Mosasaurinae (e.g., Bell, 1997; Bardet et al., 2005; Bell & Polcyn, 2005; Leblanc, Caldwell & Bardet, 2012; Palci, Caldwell & Papazzoni, 2013; Jiménez-Huidobro & Caldwell, 2016), but does not reflect the possible close connection of plioplatecarpines with yaguarasaurines (as suggested by Polcyn & Bell [2005: 332] and then inferred, together with *Tethysaurus*, by Dutchak & Caldwell [2009: Fig. 5]). Thus, we included two additional external specifiers, *Tethysaurus nopcsai* and *Yaguarasaurus columbianus*, that assure the adherence of the name Plioplatecarpinae to the traditional contents under alternative hypotheses.

The topologies inferred through our parsimony and Bayesian analyses support the monophyly of the traditional plioplatecarpines as delimited by Konishi & Caldwell (2011) (Figs. 1–7).

### Tylosaurinae Williston, 1897

#### Preferred phylogenetic definition

The most inclusive clade containing *Tylosaurus proriger* (Cope, 1869), but not *Plioplatecarpus marshii* Dollo, 1882, or *Mosasaurus hoffmannii* Mantell, 1829. This definition is branch-based.

#### Remarks

The tylosaurine interrelationships have been intensively studied during the past decade (e.g., Bullard, 2006; Martin & Fernández, 2007; Caldwell et al., 2008; Bullard & Caldwell, 2010; Jiménez-Huidobro & Caldwell, 2016; Otero et al., 2017), resulting, among others, in numerous changes in binomial nomenclature. The monophyly of Tylosaurinae, nevertheless, has not been put into question.

Conrad (2008: 130) defined Tylosaurinae as “[a]ll taxa sharing a more recent common ancestor with *Tylosaurus proriger* than with *Mosasaurus hoffmannii* or *Plioplatecarpus marshi*[*i* ]”. This definition adheres to the traditional contents of Tylosaurinae under all current topologies, including these inferred by our parsimony and Bayesian analyses (Figs. 1–7).

**Figure 8:**
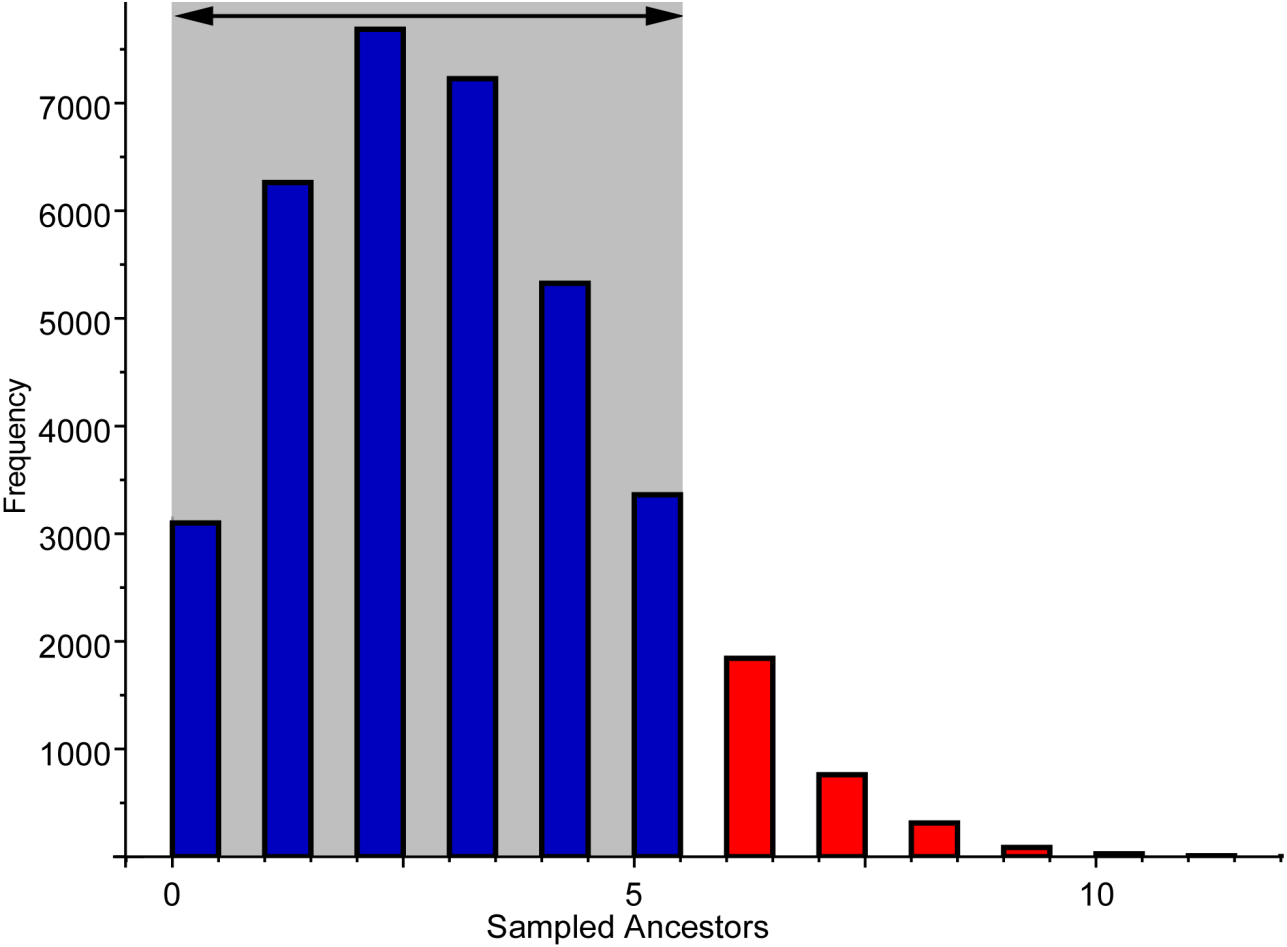
Frequency of sampled ancestors among the alternative topologies produced by the Bayesian analysis using the FBDSA model. Grey area indicates the 95% confidence interval of sampled trees.

## Discussion

### Inferences using the Fossilized Birth–Death model with sampled ancestors (FBDSA)

The FBDSA model, that discriminates between cladogenetic and anagenetic patterns in macroevolution (Gavryushkina et al., 2014; Gavryushkina et al., 2017), inferred several ancestral-descendent relationships, a subset of which is shown in the MCCT (see Fig. 5). Nevertheless, all of them were weakly supported and therefore are not discussed further. Instead of focusing on the consensus topologies (like the MCCT), a more accurate way for estimating the frequency of ancestor-descendant relationships obtained by the Bayesian analysis is by considering all the post-burnin topologies inferred (see Cau, 2017). In the 95% of the sampled trees using the data set of Simões et al. (2017), the number of sampled ancestors inferred ranges between 0 and 5 (Fig. 8), which suggests that up to 11% of the included mosasauroid taxa are potential direct ancestors of one or more other mosasauroids included. Nevertheless, these values probably overestimate the frequency of sampled ancestors. It should be remarked that in these analyses, the character list *a priori* excludes invariant characters (in particular, the autapomorphies of terminal units), as is common practice in parsimony analyses sampling exclusively potential synapomorphies. This methodological bias thus may inflate the frequency of the sampled ancestors, since it does not discriminate between actual ancestors along anagenetic lineages (that have a null terminal branch length) from spurious zero-length terminal branches due to omission of autapomorphies. In conclusion, taking into account the methodological bias due to omission of invariant characters from the morphological features included, this analysis suggests that no more than one-tenth of the inferred relationships among the actual phylogenetic tree of Mosasauroidea could be tentatively interpreted as anagenetic (direct ancestor-descendant) patterns.

### Potential issues resulting from application of the Implied Weighting function

As shown by Simões et al. (2017) and our parsimony and Bayesian analyses, the structure of the mosasauroid phylogenetic tree is highly dependent on the applied tree-search strategies. Use of some phylogenetic methods may currently lead to prefer insufficiently supported phylogenetic hypotheses. For example, Simões et al. (2017) performed a single test of parsimony analysis using the Implied Weighting (IW) function, keeping the default value for the *K* parameter (*K* = 3). Compared to their unweighted parsimony analyses, which show polytomies near the base of Mosasauroidea and within Mosasaurinae (Simões et al., 2017: Figs. 1A, 1B), the topology inferred from the parsimony analysis with IW function was fully resolved (Simões et al., 2017: Fig. 1C), and represented the only unambiguous support for a single origin of the hydropedal and hydropelvic conditions that are related to the transition from semi- to a fully aquatic lifestyle (with a reversal within Tethysaurinae to plesiopelvic condition). However, the evolutionary meaning of the *K* parameter is currently hotly debated (e.g., O’Reilly et al., 2016; Congreve & Lamsdell, 2016; Goloboff, Torres & Arias, 2017), and a recent investigation of the effects of implied weighting on modeled phylogenetic data revealed particularly poor abilities of the method to resolve data sets with large amounts of conflicts or polytomies (Congreve & Lamsdell, 2016). Goloboff, Torres & Arias (2017) criticized some aspect of the studies by O’Reilly et al. (2016) and Congreve & Lamsdell (2016) but repeated the necessity for the investigation of proper values of *K* relative to the numbers of analyzed taxa (Goloboff, 1993; Goloboff, 1995) and evaluation of more than a single concavity parameter (Goloboff et al., 2008).

It is far beyond the scope of the present paper to contribute to the debate, but given that concerns regarding the ‘proper’ use of weighted parsimony still exist, we suggest that the results of parsimony analyses with the IW function are generally treated ‘conservatively’. That is, rather than preferring a single inferred topology with a particular value of *K* that seems to fit best for the analyzed data, trees produced by different runs should be compared in order to spot, and prioritize, the groupings that are consistently being reconstructed. For example, all weighted parsimony analyses reconstruct monophyletic Halisaurinae (*Halisaurus* + *Eonatator*) but the position of this clade on the mosasauroid tree is unstable. They are either, the sister taxon to the clade formed by tethysaurines, yaguarasaurines, tylosaurines, and plioplatecarpines (Figs. 3A–3C, and 3E) or the sister taxon to mosasaurines (Figs. 3D, 3F). We suggest that regardless of which of the two hypotheses is inferred following the use of the best-fitting value(s) of *K*, the position of halisaurines should be regarded as unstable and, ideally, compared to the results produced by other methods of phylogenetic inference. Therefore, in the case of the present data set, the position of halisaurines should be treated as ambiguous. The only method that infers a strong support for either hypothesis is the Bayesian analysis that reconstructs halisaurines as the sister taxon to mosasaurines (*pp* = 0.96).

### Data sampling

Following the results of the phylogenetic analyses using multiple tree-search strategies, we discuss the factors in the data sampling that might influence the differing hypotheses of mosasauroid phylogenetic relationships and their statistical support, and suggest further changes to the explored data set that might improve the resolution of the mosasauroid phylogenetic relationships.

#### Outgroup selection

In the initial version of the data set introduced by Bell (1993) and Bell (1997), the outgroup was constructed following the algorithm described by Maddison, Donoghue & Maddison (1984). The final outgroup OTU was based on the characters present in eight modern squamates (*Aspidoscelis sexlineata*, *Crotaphytus collaris*, *Dipsosaurus dorsalis*, *Gekko gecko*, *Gerrhonotus liocephalus*, *Plestiodon laticeps*, *Shinisaurus crocodilurus*, and *Varanus niloticus*) and two extinct squamates (*Estesia mongoliensis* and *Gilmoreteius chulsanensis*). Such ‘composite’ operational taxonomic unit was used by most later authors (e.g., Bell & Polcyn, 2005; Caldwell & Palci, 2007; Leblanc, Caldwell & Bardet, 2012). More recently, however, some studies preferred to use only the character states present in *Varanus* as the outgroup (e.g., Palci, Caldwell & Papazzoni, 2013; Jiménez-Huidobro & Caldwell, 2016) “because both taxa [i.e., Mosasauroidea and *Varanus*] are large-bodied anguimorphs that share a number of symplesiomorphic features” (Palci, Caldwell & Papazzoni, 2013: 608).

The outgroup sampling is known to have a great effect on the structure of phylogenetic trees (e.g., Graham, Olmstead & Barrett, 2002; Spaulding, O’Leary & Gatesy, 2009; Kirchberger et al., 2014; Wilberg, 2015). Given the alternative placements of Mosasauroidea among different phylogenies published (e.g., Conrad, 2008; Gauthier et al., 2012; Reeder et al., 2015), it is not universally agreed which squamates may represent the closest sister group of mosasauroids. Therefore, outgroup selection among extant squamates may be biased by preference among the alternative placement of Mosasauroidea.

The problems with the use of the ‘composite’ OTU, then, was already commented on by Palci, Caldwell & Papazzoni (2013: 608) who noted that the “outgroup is problematic for several reasons: (1) it does not reflect the character state composition of a real organism; (2) it can produce paradoxical combinations of character states where a feature coded as absent in one character is further defined in a second character […]; and (3) lack of repeatability of the process that produced such codings”, noting that Bell (1997) “was not very explicit on how he obtained the character states for his outgroup”. The third point (lack of repeatability of the process), however, does not seem to be entirely fair. Even though Palci, Caldwell & Papazzoni (2013) are certainly correct that Bell (1997) was not particularly specific regarding the scores of his ‘composite’ OTU, that paper was supposed be the published version of his PhD thesis (Bell, 1993), which is explicitly referred to by Bell (1997: 294) and includes information on where the scores come from (Bell, 1993: 9–16, 251, 265–268).

To solve the issues with outgroup selection, Simões et al. (2017) expanded the data set by adding three ‘dolichosaur-grade’ taxa: *Adriosaurus suessi* Seeley, 1881, *Dolichosaurus longicollis* Owen, 1850, and *Pontosaurus kornhuberi* Caldwell, 2006, and designed *A. suessi* as the basalmost outgroup. Even though *A. suessi* constitutes a much better outgroup than the ‘composite’ OTU and *Varanus*, because its age and morphology more closely reflect those of the last common ancestor of all mosasauroids, such approach forces *Dolichosaurus* and *Pontosaurus* to be inferred more closely to mosasaurids than to *Adriosaurus*. This outgroup setting may thus lead to the construction of an artificial ‘dolichosaur grade’ as the basalmost mosasauroid condition (i.e., due to the outgroup setting in TNT used by Simões et al., 2017, ‘dolichosaurs’ are constrained to form a paraphyletic series leading to Mosasauroidea), which may lead to spurious relationships among the ingroup taxa merely based on squamate symplesiomorphies that are absent among the ‘dolichosaur’ taxa. As Simões et al. (2017) noted, some studies reconstruct these ‘dolichosaurs’ to represent snake-branch pythonomorphs (see e.g., Palci & Caldwell, 2007; Caldwell & Palci, 2010; Palci & Caldwell, 2010). Thus, all these three OTUs may be ‘equally’ distantly related to Mosasauridae. It is noteworthy that the latter hypothesis is supported by the Bayesian analysis using the FBDSA model, which reconstructed all ‘dolichosaur’ taxa as forming a clade excluding all other OTUs.

To avoid any bias due to *a priori* assumptions on character state transformation (because of the alternative extant squamate outgroup used and potentially incorrect outgroup/basal ingroup designation), we suggest to perform analyses using different outgroup selection or to consider the use of a ‘remote outgroup’. Perhaps, the well preserved Early Cretaceous (Aptian) squamate *Huehuecuetzpalli mixtecus* Reynoso, 1998, might serve as the root in a separate analysis. That taxon is universally recognized as more basal than any alternative mosasauroid outgroup used previously (Conrad, 2008; Gauthier et al., 2012), and may represent the ancestral squamate morphology regardless of the preferred closest relatives of mosasauroids. However, see also Graham, Olmstead & Barrett (2002) and Kirchberger et al. (2014) for independent tests regarding the effects of the use of phylogenetically distant outgroups in molecular studies.

#### Taxon sampling

As discussed above, the outgroup selection has a substantial impact on the structure of the inferred tree topology, including the statistical support of the basal branching near the root of Mosasauroidea. Still, the resolution of the rootward mosasauroids might not necessarily improve without an increased number of early mosasaurids and near-mosasaurids analyzed. The most recent version of the data set was expanded with the addition of *Adriosaurus suessi*, *Dolichosaurus longicollis*, and *Pontosaurus kornhuberi*, and separation of *Opetiosaurus bucchichi* from the *Aigialosaurus* OTU (even if it is assigned to *Aigialosaurus* as *A. bucchichi*; Dutchak & Caldwell, 2009; Simões et al., 2017). Still, it could also benefit, for instance, from addition of *Acteosaurus tommasinii* (Palci & Caldwell, 2010), *Adriosaurus microbrachis* (Palci & Caldwell, 2007), *Adriosaurus skrbinensis* (Caldwell & Palci, 2010), *Aphanizocnemus libanensis* (Dal Sasso & Pinna, 1997), *Carsosaurus marchesettii* (e.g., Caldwell, Carroll & Kaiser, 1995; Caldwell & Palci, 2007), *Coniasaurus crassidens* (Caldwell & Cooper, 1999), *Eidolosaurus trauthi* (Nopcsa, 1923), and *Pontosaurus lesinensis* (Pierce & Caldwell, 2004). The fact that some or most of these taxa can be more closely related to snakes than to mosasaurids (see e.g., Palci & Caldwell, 2007; Caldwell & Palci, 2010; Palci & Caldwell, 2010), is not a problem as their morphology approximates to that of the mosasaurid ancestor, and therefore supplements the knowledge of early pythonomorph evolution.

The data set of Simões et al. (2017) contains members of all well-recognized mosasauroid subclades; the taxa traditionally contained within Halisaurinae, Mosasaurinae, Plioplatecarpinae, and Tylosaurinae. It also contains all tethysaurines and yaguarasaurines (except *Romeosaurus sorbinii* Palci, Caldwell & Papazzoni, 2013), as these two clades were inferred in studies using recent versions of the data set (Makádi, Caldwell & Ösi, 2012; Palci, Caldwell & Papazzoni, 2013; respectively). Still, some of the clades are substantially underrepresented even though detailed descriptions of their members have been published and some of those taxa have been scored for characters in older versions of the same data set. For example, the current version of the data set includes only two halisaurine OTUs (*Halisaurus platyspondylus* and *Eonatator sternbergii*; with the latter being labeled as ‘*Halisaurus sternbergi*’) even though detailed studies have also been published, for example, for *Halisaurus arambourgi* (Bardet et al., 2005; Polcyn et al., 2012) or *Phosphorosaurus ortliebi* (Lingham-Soliar, 1996; Holmes & Sues, 2000; Bardet et al., 2005). Likewise, the data set could be supplemented by recently described *Eonatator coellensis* (Páramo-Fonseca, 2013) and *Phosphorosaurus ponpetelegans* (Konishi et al., 2016). Such sampling could test some of the implied relationships (the connection of *E. coellensis* to *E. sternbergii*, *H. arambrourgi* to *H. platyspondylus*, *P. ponpetelegans* to *P. ortliebi*). A phylogenetic analysis of Halisaurinae was recently published by Konishi et al. (2016). The analysis did not reconstruct monophyletic *Halisaurus* nor *Eonatator* but inferred sister-taxon relationships between *P. ortliebi* and *P. ponpetelegans*, a taxon described by these authors. However, the analysis was based on only 21 cranial characters and rooted on *Platecarpus tympaniticus*, a derived plioplatecarpine that might not serve best as the outgroup for such analysis due to its placement and age. Considering the unsettled relationships within Halisaurinae, and the differing position of the clade within Mosasauridae, an expansion of the data set by using more halisaurines (and modification of the characters to better reflect their morphology) might result in improving the resolution of the mosasauroid tree topology.

New reappraisals of certain tylosaurine species have also been published recently. For example, *Hainosaurus pembinensis* and *H. bernardi*, the latter being the type species of *Hainosaurus*, have been assigned to *Tylosaurus* (Bullard & Caldwell, 2010; Jiménez-Huidobro & Caldwell, 2016, respectively), and *Tylosaurus kansasensis* was proposed to be a juvenile of *T. nepaeolicus*, and thus removed from the data set (Jiménez-Huidobro, Simões & Caldwell, 2016). However, *T. pembinensis* is not included in the recent version of the data set, which does not enable to further test the newly proposed hypotheses. Interestingly, the ordered-unweighted parsimony analysis and the Bayesian analysis do not support the monophyly of *Tylosaurus* (represented by *T. proriger*, *T. bernardi*, and *T. nepaeolicus*) exclusive of *Taniwhasaurus* (Figs. 2 and 5). When only one ‘dolichosaur’ is in the data set and used as the outgroup, regardless of which one it is, *Tylosaurus* is monophyletic (Fig. 4). The resolution might improve with a more appropriate outgroup selection and addition of *T. pembinensis* and, possibly, ‘*Hainosaurus*’ *neumilleri* (Martin, 2007). Additionally, *Tylosaurus* ‘*saskatchewanensis*’ (Bullard, 2006) and ‘*Hainosaurus*’ ‘*kenbrowni*’ (Thompson, 2005; Thompson, 2011) can also be considered pending their formal descriptions.

The understanding of the plioplatecarpines, in turn, may improve by separation of the *Plioplatecarpus* OTU into several terminal units. Such sampling could test the monophyly of *Plioplatecarpus* (a taxon consisting of a few species, including *P. marshii*, *P. houzeaui*, *P. primaevus*, and the recently described *P. peckensis*; Cuthbertson & Holmes, 2015), estimate the support for the tree topology obtained by Konishi & Caldwell (2011) and Cuthbertson & Holmes (2015), test the connection of ‘*Latoplatecarpus*’ *nichollsae* and *L. willistoni*, or provide additional support for the separation of *Plesioplatecarpus planifrons* (labeled as ‘*Platecarpus planifrons*’ in the data set of Simões et al., 2017) from *Platecarpus tympaniticus* (Konishi & Caldwell, 2011).

Mosasaurines are problematic, as is apparent from differing and often poorly resolved tree topologies. The inference of the structure of the mosasaurine phylogenetic tree appears to be difficult especially due to the unstable positions of the taxa attributed to *Prognathodon* (e.g., Leblanc, Caldwell & Bardet, 2012; Simões et al., 2017; our study). Nevertheless, numerous derived mosasaurines are currently under revision as is apparent from Street & Caldwell (2017) that provided detailed reappraisal of *Mosasaurus hoffmannii*, preliminary discussion of some other taxa traditionally assigned to *Mosasaurus*, and reported on an ongoing research. Together with reconsideration of some species traditionally attributed to *Prognathodon*, the resolution of the mosasaurines might benefit from addition of some presumably rootward mosasaurine taxa that have not been included in previous ‘complete’ versions of the Bell’s data set (i.e., when the aim was to assess the interrelationships within all major clades of mosasauroids). These include, for example, *Kourisodon puntledgensis* (Nicholls & Meckert, 2002). This taxon, which has previously been used as an outgroup in some analyses (Konishi & Caldwell, 2011; Cuthbertson & Holmes, 2015), originates from the upper Santonian of British Columbia, Canada, and is one of the oldest known mosasaurines. Its inclusion might have an impact on the resolution of Mosasaurinae.

#### Character sampling

We suggest that character statements are redefined from those used in recent versions of Bell’s (1997) data set, following the recommendations in Sereno (2007) and Brazeau (2011). In particular, compound characters are suggested to be atomized; i.e., neomorphic and transformational features should be considered as distinct characters and not as alternative states of a single character. Therefore, when not resulting in loss of information, characters are suggested to be defined as binary. When multistate character statements are included, and the states form unambiguous morphoclines that describe a nested set of alternative states (e.g., marginal tooth numbers, vertebral numbers, phalangeal formulas), the corresponding character statements should be set as ordered, to avoid *a priori* exclusion of potential synapomorphies represented by the subset of states representing a derived condition (e.g., Wilkinson, 1992; Sereno, 2007; Brazeau, 2011). Such states, however, should be formulated to avoid marked polymorphism. For example, the current version of the data set (Simões et al., 2017) includes a six-state character dealing with the dentary tooth count: “(53) Dentary tooth number: 20–24 (0); 17–19 (1); 15–16 (2); 14 (3); 13 (4); 12 (5)”. Yet such defined states insufficiently reflect differences in taxa where the dentary tooth count is one of the few distinguishing characters. Furthermore, once set as ordered to reflect the homology among nested state-transitions, the character defined this way leads to inflating the phylogenetic importance of a feature that may be merely size-related and individually variable among the same taxon. For instance, *Mosasaurus hoffmannii* is often reported as having 14 dentary teeth (e.g., Street & Caldwell, 2017). However, some specimens have 15 dentary teeth (e.g., CAMSM F22228, IRSNB R 0303; D Madzia, pers. obs., 2017; Mulder, Cornelissen & Verding, 2004), or only 13 (NHMM 009002; Everhart et al., 2016). Thus, *M. hoffmannii* can be scored for states 2, 3, and 4. At the same time, *Mosasaurus lemonnieri*, which is currently considered to be distinct from *M. hoffmannii* (Street & Caldwell, 2017; D Madzia, 2017, unpublished data), has always 16 dentary teeth. Still, it would be covered under the same state (2).

This example demonstrates that character definitions and among-state transition settings may significantly influence relationships, and must be discussed prior to phylogenetic analyses.

#### ‘Data handling’

As we have expressed above, we consider the current versions of the Bell’s (1997) data set to be insufficient for accurate inferences of mosasauroid phylogenetic relationships. We suggest to (1) reconsider the outgroup selection, (2) increase the number of analyzed taxa, and named some of those that we think might improve the resolution of the mosasauroid phylogenetic tree, and (3) revise the morphological characters and their states. Naturally, it is essential to note that the steps should be undertaken after careful considerations and simultaneously. Specifically, increasing the number of analyzed taxa could have an entirely opposite effect and cause more instability if the additions do not sufficiently reflect the differing morphologies of the proposed OTUs and their character evolution. Also, we suggest to consider even those taxa that might be regarded as too incomplete to be included in the data matrix (see e.g., Wiens, 2003a; Wiens, 2003b; Wiens & Morrill, 2011). The relevance of all additions might be tested, for example, following the principle of safe taxonomic reduction (Wilkinson, 1995), using TAXEQ3 (Wilkinson, 2001), or through ‘concatabominations’ (Siu-Ting et al., 2015). However, it has also been argued that “there is no justification—either *a priori* or *a posteriori*—to definitively exclude unstable taxa from the data matrix as this involves the deletion of phylogenetic information that can be relevant (or even critical) for understanding the relationships of the entire group” (Pol & Escapa, 2009: 13). Therefore, Pol & Escapa (2009) offered to use a TNT script, IterPCR, that provides a list of characters related to the instability of each unstable taxon. This script has already been implemented in TNT (Goloboff & Szumik, 2015).

## Conclusions

Throughout the last two decades the phylogenetic relationships within Mosasauroidea have been inferred using modified versions of a single data set, originally published by Bell (1997). In order to estimate the robustness in our understanding of mosasauroid phylogenetic relationships, we used a recent version of that data set (published by Simões et al., 2017) and focused on the effects of tree-search strategy selection.

Parsimony and Bayesian analyses of the same data set showed considerable differences in tree topologies near the base of Mosasauroidea, suggesting that an increased number of the basal taxa and morphological characters phylogenetically informative for large-scale relationships need to be taken into account. Furthermore, the different topologies obtained by the alternative tree-search strategies suggest that one particular phylogenetic hypothesis may be significantly biased by the phylogenetic method used as suggested by Simões et al. (2017). We thus suggest to perform different analyses of the same data, using alternative tree-search strategies and tree models, and to consider as supported only those hypotheses shared consistently by the majority of analyses. Following the results of the present study, the monophyly of the traditional mosasauroid groups (Halisaurinae, Tethysaurinae, Plioplatecarpinae, Tylosaurinae, Mosasaurinae, and possibly also Yaguarasaurinae) can be currently considered supported. Yet their mutual relationships as well as the relations within these groups are still largely unsettled.

From the nomenclatural perspective, we see little or no support for the use of some binomial combinations. Specifically, our analyses often failed to reconstruct monophyly for the mosasaurine taxon *Prognathodon*. Although the Bayesian analysis infers some support, albeit extremely poor, for a clade formed by all taxa attributed to *Prognathodon* (and including *Eremiasaurus*), ‘*Prognathodon*’ requires complex reassessment and some taxa will have to be removed from it (see also, e.g., Leblanc, Caldwell & Bardet, 2012; Simões et al., 2017).

We recommend that future implementations of the mosasauroid data set will discuss the combined effects of taxon sampling, character construction, and tree-search strategy settings. For instance, in phylogenetic analysis using parsimony, and where all characters are set as having equal weight, the splitting of the multistate characters into distinct binary characters does not bias the reconstruction of the state transitions. On the contrary, in phylogenetic analysis using parsimony as tree-search strategy, and with the Implied Weighting function, multistate or compound characters, once subdivided into binary characters, are analyzed with different weighting settings. Furthermore, in Bayesian phylogenetic analyses where rate variation across morphological characters are modeled using the gamma parameter, different state transitions of the same morphocline may evolve at different rates.

We conclude that, until the data set is significantly improved by a more appropriate taxon sampling and revision of characters, the currently inferred phylogenetic relationships of mosasauroids should be seen as tentative and subject to change.

##  Supplemental Information

10.7717/peerj.3782/supp-1 Supplemental Information 1 TNT file for parsimony analyses

10.7717/peerj.3782/supp-2 Supplemental Information 2 BEAST file for Bayesian analysis

## Institutional abbreviations

CAMSM: Sedgwick Museum of Earth Sciences, University of Cambridge, Cambridge, UK
IRSNB: Royal Belgian Institute of Natural Sciences, Brussels, Belgium
NHMM: Natuurhistorisch Museum Maastricht, Maastricht, the Netherlands.

## Additional Information and Declarations

### Data Availability

The following information was supplied regarding data availability: The raw data has been supplied as a Supplementary File.
